# Distinctive Effects of Cytochalasin B in Chick Primary Myoblasts and Fibroblasts

**DOI:** 10.1371/journal.pone.0154109

**Published:** 2016-04-27

**Authors:** Koichi Ojima, Zhong-Xiang Lin, Ivone Rosa de Andrade, Manoel Luis Costa, Claudia Mermelstein

**Affiliations:** 1 Animal Products Research Division, NARO Institute of Livestock and Grassland Science, Tsukuba, Ibaraki, 305–0901, Japan; 2 Department of Cell Biology, Beijing Institute for Cancer Research, Beijing Medical University, Beijing, 100083, China; 3 Instituto de Ciências Biomédicas, Universidade Federal do Rio de Janeiro, Rio de Janeiro, RJ, 21941–902, Brasil; Institut de Myologie, FRANCE

## Abstract

Actin-based structures play fundamental roles in cellular functions. However it remains controversial how cells cope with the absence of F-actin structures. This report focuses on short- and long-term effects of cytochalasin B (CB) on actin-complexes in fibroblasts and myoblasts. Thirty min of CB treatment dispersed subplasma actin cortices, lamellipodia, ruffled membranes, stress fibers and adhesion plaques into actin patches in fibroblasts and muscle cells. In contrast, 72 hrs CB treatment showed distinct morphological effects. Fibroblasts became giant multinucleated-finger shaped with 5 to 10 protrusions, 3–8 μm in width, and >200 μm in length. They lacked cortical actin, stress fibers, adhesion plaques and ruffled membranes but contained immense lamelliopodia with abnormal adhesion plaque protein complexes. Muscle cells transformed into multinucleated globular-shaped but contained normal I-Z-I and A-bands, indicating that CB did not interfere with the assembly of myofibrils. Within 30 min after CB removal, finger-shaped fibroblasts returned to their original shape and actin-containing structures rapidly reappeared, whereas muscle cells respond slowly to form elongated myotubes following CB washout. The capacity to grow, complete several nuclear cycles, assemble intermediate filaments and microtubules without a morphologically recognizable actin cytoskeleton raises interesting issues related to the role of the actin compartments in eukaryotic cells.

## Introduction

In many cell types the conserved, actin-based cytoskeleton consists of 5 kinetically labile sub-structures, each subject to rapid changes. They are the (1) lamellipodia, (2) ruffled membranes, (3) stress fibers, (4) adhesion plaques and (5) subplasma actin cortices. Over the past decades numerous studies have focused on the regulatory molecules controlling the assembly/disassembly of the first 4 sub-structures and how they mediate directional locomotion. The outcome of this work has been the delineation of complex interactions between many signaling and structural molecules. Early studies emphasized the upstream activity of Rho, Rac, and Cdc42 in regulating the assembly, respectively, of stress fibers and adhesion complexes, lamellipodia and/or ruffled membranes and filopodia [1]. These in turn activate formins, WASP, Scar/Wave, and still more downstream molecules like ERM, Arp2/3 and associated proteins [2–7]. These findings have converged with elegant immuno-electron microscopy observations demonstrating that the Arp2/3 complex nucleates and caps actin monomers at the sides of pre-existing filaments which subsequently elongate by their barbed ends [8–11]. These complementary findings have provided a compelling molecular/structural overview suggesting how branched F-actin networks might drive translocating lamellipodia, as well as leading to the assembly of adhesion complexes and their associated stress fibers.

The challenge of how coordinated interactions between these 4 actin sub-structures regulate cell migration has tended to eclipse efforts to understand the assembly and function of the remaining major cytoskeleton component, namely the subplasma cortical actin lattice. In erythrocytes, where it has been studied most intensively, the plasma lipid bilayer is supported by a cortex consisted by a network of short actin/tropomyosin filaments capped at their pointed ends and barbed ends by tropomodulin and adducins, respectively [12]. These in turn are cross-linked into a planar hexagonal lattice by flexible isoforms of spectrin [12–14]. The subplasma actin/spectrin network, in conjunction with a variety of transmembrane molecules, conforms in outline with the juxtaposed plasma membrane proper [15].

Primary embryonic chick myogenic cultures consist of approximately equal numbers of precursor replicating fibrogenic and myogenic cells. It is an important tool to address the role of different actin compartments because these two cell types will assemble quite different actin structures upon cell differentiation. Both cell types display actin-based cytoskeletal structures including prominent subplasma actin/spectrin cortices. Previous studies [16–18] reported that while some of the actin based structures in these fibrogenic and myogenic cells responded similarly to the inhibitor of actin polymerization cytochalasin B (CB), others responded in a cell-specific manner. For example, while blocking cytokinesis, but not nuclear duplication, CB did not block the terminal differentiation of myogenic cells and accumulation of either fibrogenic or myogenic cells. One consequence of these effects was that after growing for 72 hrs in CB these cultures displayed 2 distinct multinucleated phenotypes. One was a giant, flattened vimentin-positive arborized cell termed a “finger-shaped fibroblast” (see below). The other, a globular, desmin-positive cell that was clearly myogenic, for it assembled I-Z-I bands of polarized 1.0 μm thin filaments that interdigitated with A-bands. These globular cells contracted spontaneously and occasionally. The limitations to these early studies, however, were both methodological and conceptual. These experiments were performed before the advent of fluorescent phalloidin [19] and confocal microscopy, as well as before actin isoforms were discovered [20]. As a consequence, many of the effects of CB on the multiple actin containing structures in both fibrogenic and myogenic cells were missed.

The current availability of fluorescent phalloidin plus many new antibodies to cytoskeletal and myofibrillar proteins, together with confocal microscopy techniques, prompted us to re-asses the short- and long-term, as well as the strikingly reversible, effects of CB on the actin based structures in cells in these primary chick myogenic cultures. Here we show that after short-term treatment with CB, both fibroblasts and myogenic cells lose their filamentous cytoplasmic and membrane-actin structures in a very similar way. By contrast, long-term CB treatments lead to distinct morphological impact on both cells, *i*.*e*. finger-shaped fibroblasts and mushroom-like myogenic cells. CB-treated fibrogenic cells, despite lacking for 3 consecutive days a morphologically detectable filamentous actin-cytoskeleton, perform a variety of basic functions. They repeatedly cycle through G1, S, G2, prophase and metaphase forming giant, multinucleated, finger-shaped fibroblasts that display enlarged but normal microtubule- and vimentin intermediate filaments-networks. Following CB-washout, these multinucleated fibroblasts assemble a normal complex actin cytoskeleton. In contrast to fibroblasts, the mushroom-shaped globular myogenic cells are myofibrillar protein positive and contain mono-, bi-, or 4^+^-nuclei. These myogenic cells contract spontaneously and occasionally and show normal I-Z-I-bands interdigitating with normal A-bands, indicating that CB do not interfere with the assembly of myofibrils but did interfere with their elongation mechanisms. We also detect atypical distribution of microtubule and intermediate filament structures after long-term CB treatment, which may be caused by the mechanical modifications induced by the broken down of actin networks.

## Results

### Effects of 30 min CB treatment on fibroblasts and muscle cells

**Fig 1:**
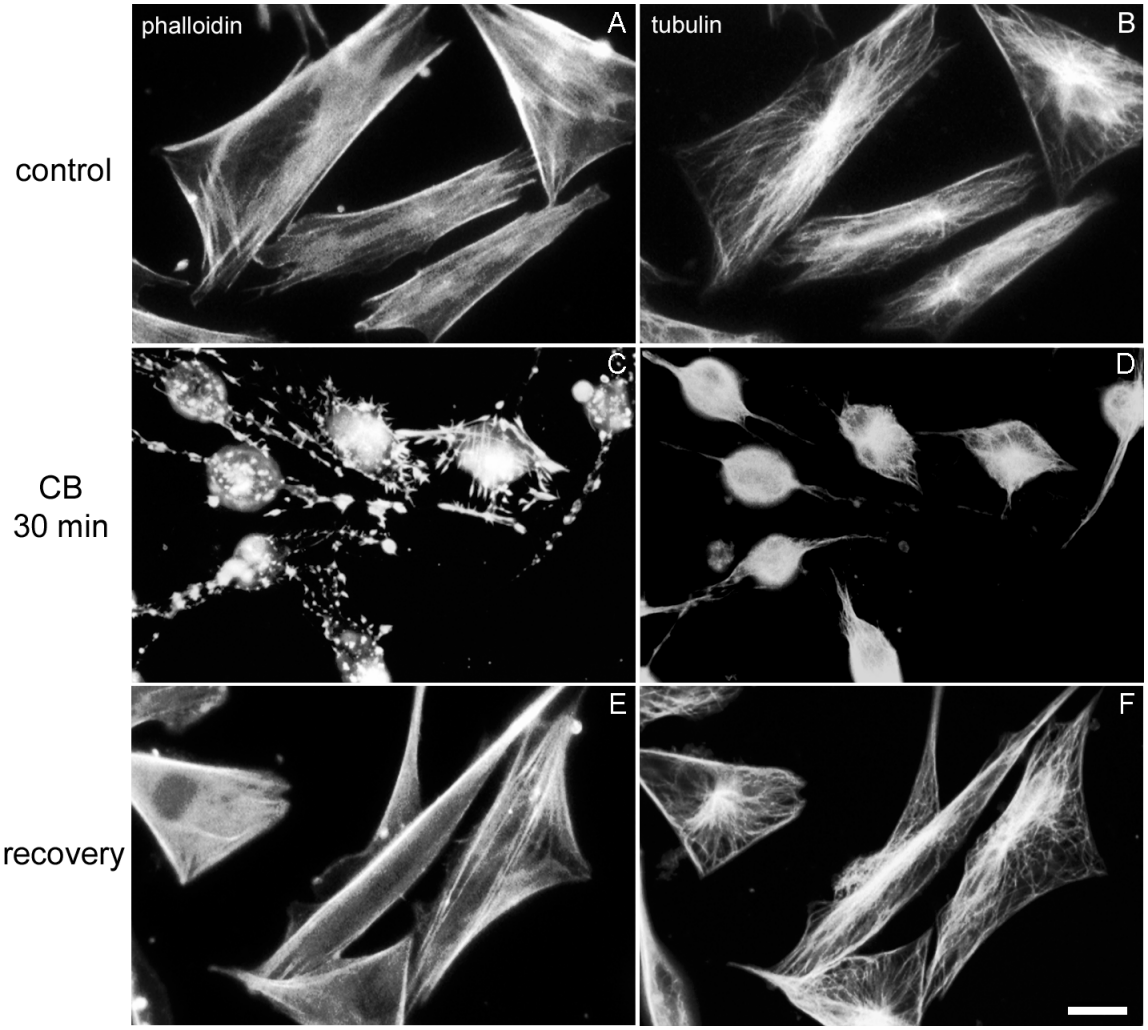
Effects of short-term CB treatment on fibroblasts. Immunofluorescence microscopy images of control chick fibroblasts (**A** and **B**), 30 min CB treated cells (**C** and **D**), and cells allowed to recover for 30 min following 30 min CB-treatment (**E** and **F**) are shown. Cells were visualized with Rho-phalloidin (**A**, **C** and **E**) and with anti-tyrosylated-α-tubulin antibody (**B**, **D** and **F**). Note that sub-plasma membrane actin cortices and multiple bundles of stress fibers are observed in the spread immobile control-untreated cells with triangular/polygonal outlines (**A** and **B**) and in the cells treated with 30 min CB and allowed to recover (**E** and **F**), but not in CB treated cells (**C** and **D**). Bar, 10 μm.

**Fig 2:**
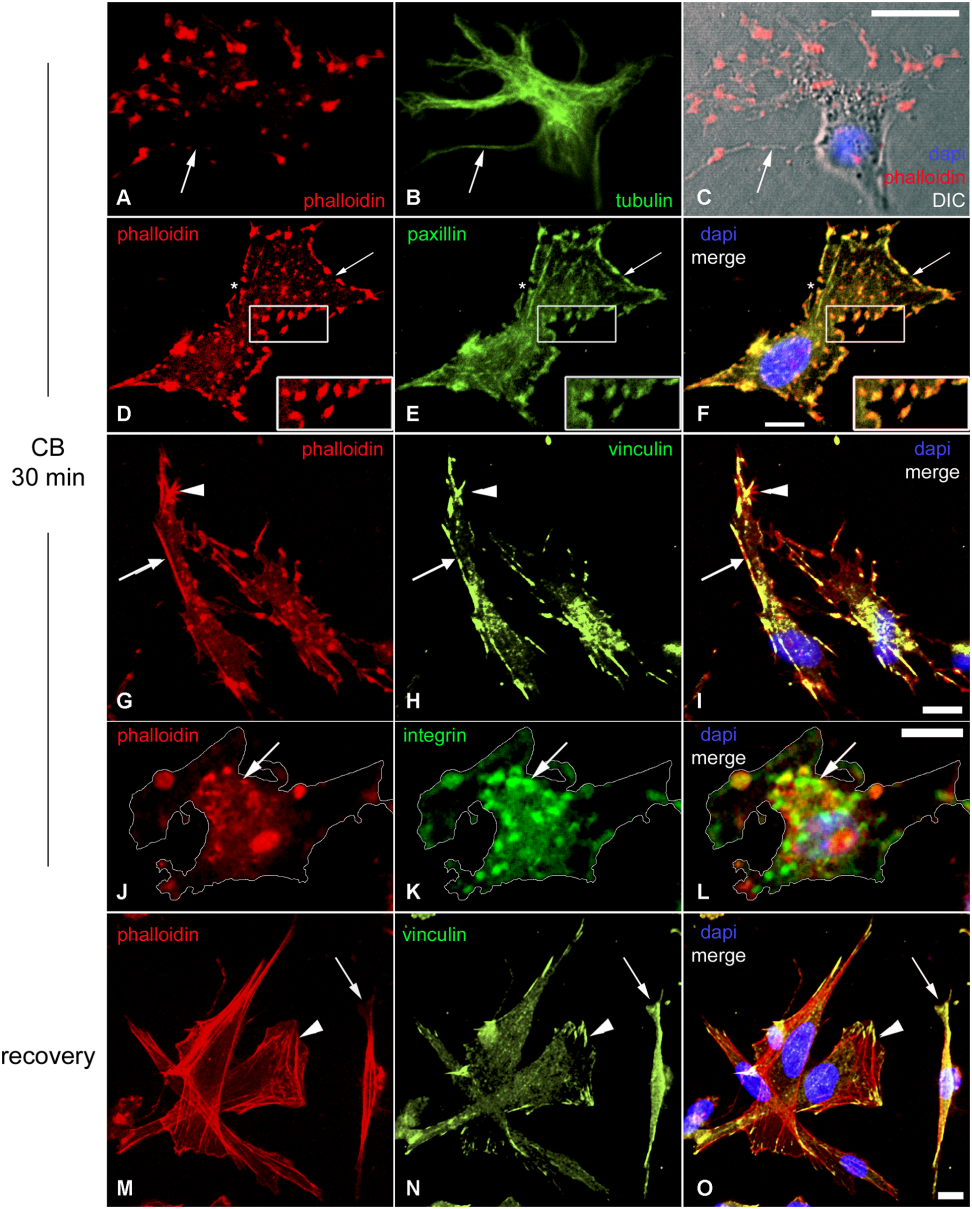
Localization of adhesion plaque proteins in short-term CB treated fibroblasts and recovery from 30 min CB treatment. Confocal microscopy images of chick fibroblasts in the presence of CB for 30 min (**A**-**L**) and subsequent recovery in normal media for 30 min (**M**-**O**) are shown. Cells were probed with Rho-phalloidin (**A**, **D**, **G**, **J** and **M**), and with antibodies against tyrosylated-α-tubulin (**B**), paxillin (**E**), vinculin (**H** and **N**) and β1-integrin (**K**). Figures **C**, **F**, **I**, **L** and **O** are superimposed images of Rho-phalloidin (red) and antibody staining (green) combined with DAPI (blue), and, in the case of **C**, differential interference contrast (grey). Arrows in **A**, **B** and **C** point to a fine and long cytoplasmatic extension where no cortical actin structure but microtubule bundles are observed. Arrows in **D**, **E** and **F** point to the absence of F-actin staining in the border of the cell, whereas the inset stresses the co-localization of actin and paxillin. Arrows in **G**, **H**, and **I** indicate continuous cortical actin that is discontinuous for vinculin whereas the arrowhead shows that the two stains are not perfectly co-incident. Arrows in **J**, **K** and **L** points to the co-localization of F-actin and β1-integrin; the outline of the cell was drawn based on the DIC image. Arrowheads in **M**, **N** and **O** indicate Rho-phalloidin negative but vinculin positive adhesion plaque structure. Arrows in **M**, **N** and **O** show a putative myoblast. Scale bars, 10 μm.

Since these day-2 (the next day of plating) muscle cell cultures are composed of young myoblasts (~50%) and mesenchymal stem cells (~50%), normally the post-mitotic myoblasts are going through myofibrillogenesis including cell fusion to develop multinucleated myotubes in the coming 2–3 days while the presumptive myoblasts need yet to complete their last 1–3 mitosis before start myofibrillogenesis. On the other hand, mesenchymal stem cells are actively mitotic to produce most of the fibroblasts. We used CB to disrupt F-actin and to study actin-containing structures of these day-2 muscle cell cultures. Firstly, we focused on the short-term treatment on fibroblastic cells. A 30 min treatment with CB was sufficient to round up fibroblasts and muscle cells (compare Fig 1A and 1B with Fig 1C and 1D). Morphologically both cell types were indistinguishable without probing with antibodies. Preliminary observations suggested that the response to CB depended on whether the fibroblastic cells were exposed for 15 or 30 min, or grown continuously in CB for 72 hrs. While a 15 min exposure to CB totally dispersed the stress fibers, adhesion plaques, lamellipodia and ruffled membranes, short fragments of the cortical actin rim often remained (not shown). However, total elimination of the cortical actin rim, which may constitute 60–90% of their perimeter in control spread cells (Fig 1A and 1B), required 30 min CB exposure (Fig 1C and 1D). Concomitant with the fragmentation of all actin-containing structures, numerous scattered actin aggregates or patches emerged throughout the cell (Fig 1C), whereas microtubules seemed to form tightly fasciculate bundles in CB treated fibroblasts (Fig 1D). These actin-containing structures varied in their sizes (~3.0 μm) and in their numbers (20-50/cell, n = 100 cells from 3 different experiments, significantly different from untreated cultures, p<0.05). Some were irregular in shape, others more crystalloid. Each patch was precisely stained with Rho-labeled phalloidin. Importantly, despite the displacement of actin from its normal sub-plasma membrane location, under the light microscope the physical integrity of the sub-plasma membrane seems to be maintained (Fig 2C). Furthermore, the displacement of F-actin complexes from the plasma membrane did not obviously impair the selective permeability or the functions of the treated cells, *e*.*g*. roughly only 3% of both control-untreated and treated cells were stained with trypan blue (n = 100 cells from 3 different experiments). Thus, we can conclude from the trypan blue exclusion assay experiments that the disorganization of actin filaments by CB did not impair the selective membrane permeability of the cells. The most convincing evidence for the apparently normal metabolic activity of cells lacking detectable actin-based cytoskeletal structures is illustrated in Fig 1C, 1D, 1E and 1F. Cells that lacked visible actin-based cytoskeleton structures just prior to CB-washout assembled one that was indistinguishable from control-untreated cells (compare Fig 1A and 1B with Fig 1E and 1F).

Given that actin-based cytoskeletal structures play roles in multiple functions and have both a complex molecular composition and global distribution, the implosion of the actin-based cytoskeleton by CB raises interesting issues: (1) the fate of molecules, other than actin, in such treated cells, (2) the molecular composition of the actin patches, which can be particularly relevant to their functions, and (3) the metabolic options of fibroblasts lacking morphological actin cytoskeletal structures for short periods (*e*.*g*. 30 min of CB treatment) versus long periods (*e*.*g*. 72 hrs of CB treatment).

In a detailed immuno-electron microscopy analysis, coupled with decorating actin filaments with myosin subfragment 1, Verkhovsky and colleagues [21] have reported that in rat fibroblasts CB-induced patches consisted of clustered actin filaments whose barbed ends oriented towards and converged on a myosin II core. Their electron microscopy images encouraged us to further investigate what type of proteins appeared in actin patches under fluorescent light microscopy. Specifically, we asked whether actin patches were heterogeneous or homogeneous in terms of composition, and if the former which proteins might be packed into an actin patch. To this end, CB-treated cultures were double-stained with Rho-phalloidin and one of the following antibodies to adhesion plaque proteins: paxillin, vinculin, or β1-integrin (Fig 2A–2L). Seventy-eighty percent of the F-actin patches in each of the 4 sets of cultures (n = 100 cells from 3 different experiments) were positive for the respective adhesion plaque proteins. Obviously, the CB-induced actin patches were not promiscuous traps for non-specifically binding of other major cytoplasmic proteins such as tubulin (Figs 1D and 2B).

Interestingly, after a 30 min treatment with CB many β1-integrin positive-intracellular vesicles were observed at the ventral surface (where the cells are attached to the substrate) of the fibroblastic cells (Fig 2J–2L). These vesicles also contained F-actin, since they were positive for phalloidin (Fig 2J–2L). β1-integrin was found in these intracellular vesicles as well as in the F-actin patches, together with other adhesion plaque proteins, such as talin, vinculin and paxillin.

### Reassembly of the actin-containing structures in fibroblasts after recovery from 30 min CB

The kinetics and sequence of transitory macromolecular structures during recovery was followed by growing CB treated cells in normal medium for 5 and 15 min after CB-washout. While many cells in the 5 min recovery series were shapeless, unexpectedly 55% (n = 100 cells from 3 different experiments) displayed the overall triangular/parallelogram morphology common to many well-spread immotile fibroblasts (Fig 2M–2O). At all stages of recovery F-actin structures were always detected with other associated proteins, such as vinculin (Fig 2M–2O). The earliest recognizable reconstituted F-actin structures at this early stage were the subcortical actin lattices. In 30 min recovered cells, as in control-untreated cells, adhesion plaques rarely associated with the actin cortex but were largely confined to the distal tips of stress fibers (Fig 2M–2O).

Reliable criteria distinguishing lamellipodia from ruffled membranes have yet to be established. Our findings show that there were significant behavioral and structural differences between the two. Movement in real time of the free edge of the ruffled membrane was readily followed by eye under the phase microscope as a lighting high contrast sharp line. After staining with phalloidin the free edge appears as an intensely fluorescent curlicue-like structure. It did not contact the substrate, did not display adhesion complexes, often extended more than 12 μm into the medium, and during recovery appears earlier than lamellipodia (Table 1). The majority of the actin containing structures that assembled 30 min after CB-washout was indistinguishable from control-untreated cells (Fig 2M–2O and Table 1). The two earliest actin sub-structures to reform concurrent with the disappearance of the ectopic actin patches were the actin cortices and ruffled membranes (Table 1). Despite the fact that ruffled membranes were one of the earliest structures to reappear after CB-washout, their presence decreased with time and only 13% of them were found after 30 min CB-washout (Table 1). Ruffled membranes are important in the initial events that occur during the cytoplasmic remodeling and the establishment of polarity in motile cells, such as fibroblasts. The decrease in number of ruffled membranes during the recovery from CB can be explained by the increase in stress fibers and adhesion plaques as the cells became more adherent and less motile.

**Table 1:** Emergence of actin cytoskeletal subunits after CB washout. Temporal reconstitution of cytoskeletal subunits in cultures exposed to CB for 30 min and then after washout permitted to recover for varying times in normal medium. In each of the 6 series, 3 dishes were double-stained at the indicated times. The first randomly selected 100 cells/dish at each time point were categorized into the different substructures.

% of Recovery time from CB washout (min)			
	5	15	30
sub-plasma actin cortex	82	100	100
adhesion plaques	53^#^	84^#^^	100^#^^
ruffled membranes	46	57	13
individual stress fibers	0	23	100
lamellipodia	0	0*	39*
sub-plasma spectrin cortex^§^	100	100	100

^#^ associated with cortical actin and not with stress fibers;

^ associated with the tips of stress fibers and not with cortical actin;

* lamellipodia which were less than 1.0 μm in width;

^§^ spectrin-positive sub-plasma cortex.

In summary, (1) the rapid sequential reassembly of the actin cytoskeleton after CB-washout involved transitory combinations of actin-associated proteins that were lost with further completion; (2) the two earliest actin sub-structures to reform concurrent with the disappearance of the ectopic actin patches were the actin cortices and ruffled membranes (Table 1).

### Effects of 30 min CB treatment on muscle cells and recovery

In order to compare fibroblasts with muscle cells, we studied the short-term treatment of CB in myoblasts. A 30 min treatment with CB was sufficient to round up muscle cells (S1 Fig). Myoblasts treated for 30 min with CB lost their cortical actin and their stress fiber-like structures while the cells round-up. Many α-actin/s-α-actinin patches were observed at the surface of these rounded myoblasts. The α-actin/s-α-actinin patches were also positive for adhesion plaque proteins, such as vinculin and paxillin. When recovered in normal medium, these CB treated-myoblasts reassembled their actin-containing filament structures in a way that cells were indistinguishable from control-untreated cultures (data not shown). Interestingly, these results showed that the short-term treatment of CB induced similar changes and recovery in the actin containing structures in both fibroblasts and myoblasts.

### Distinct morphological effects of 72 hrs CB treatment on fibroblasts and muscle cells

**Fig 3:**
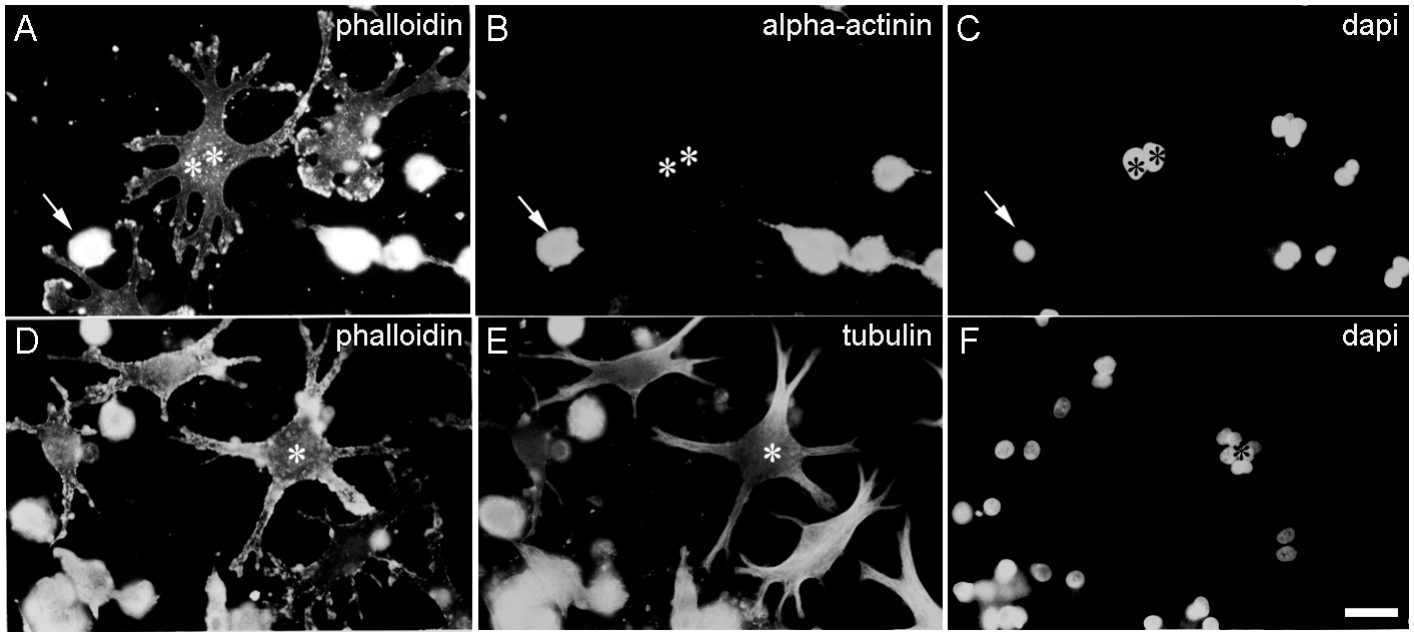
Effects of long-term CB treatment on fibroblasts and myoblasts. Immunofluorescence microscopy images of chick fibroblasts and myoblasts that were treated with CB for 72 hrs are shown (**A**-**F**). Cells were stained with Rho-phalloidin (**A** and **D**), with an antibody against sarcomeric-α-actinin (**B**), with an anti-tyrosylated-α-tubulin antibody (**E**) and with DAPI (**C** and **F**). Note that only round myoblasts are positive for s-α-actinin in **B** (arrows in **A**-**C**) but the bi-nucleated fibroblastic cell is negative for s-α-actinin (double asterisks in A-**C**). Tetra-nucleated finger-shaped fibroblasts are indicated by asterisks (**D-F**). Bar, 20 μm.

**Fig 4:**
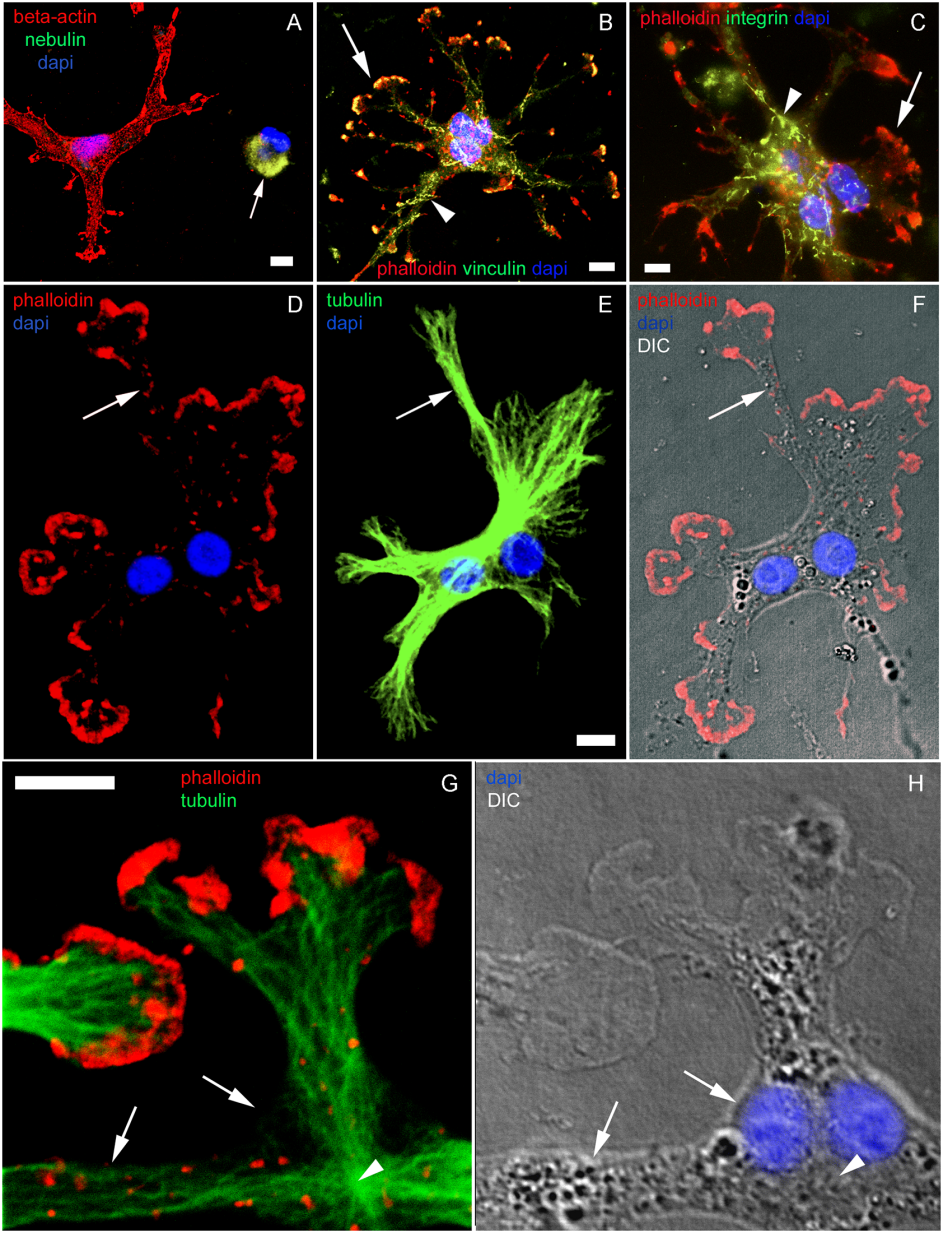
Effects of long term CB treatment on fibroblasts. Day 2 chick fibroblasts were treated with CB for 72 hrs (**A**-**H**), and subsequently cells were probed with Rho-phalloidin (red in **B**, **C**, **D**, **F** and **G**) and antibodies to β-actin (red in **A**), nebulin (green in **A**), vinculin (green in **B**), β1 integrin (green in **C**), and tyrosylated-α-tubulin (green in **E** and **G**). Figures **A**, **B** and **C** are merged images of the double antibody staining combined with DAPI (blue). Figures **D**, **E** and **F** are the same field and DIC (grey) is shown in **F**. Contrast in image **E** has been enhanced to allow a better visualization of isolated MTs filaments at the periphery of a finger-shaped fibroblast. High magnification images of lamellipodia and cytoplasmic extension in finger-shaped fibroblasts are shown in **G** and **H**. Figure **G** and DIC image in figure **H** are the same field. Note that distinct effects of long term CB treatment on fibroblasts and myogenic cells are observed, *i*.*e*. fibroblasts change to finger-shaped cells with abnormal distribution of focal adhesion plaque proteins which are termed filigree (arrowheads in **B** and **C**) whereas myogenic cells turn to round shape (arrow in **A**). Arrows in **B** and **C** indicate lamellipodia. Arrows in **D**, **E** and **F** point to a fine cytoplasmic process in which bundles of dense microtubules run through. Arrows in **G** and **H** indicate plasma membrane without detectable microfilaments and microtubules. Arrowheads in **G** and **H** point to a microtubule-organizing center close to the nuclei of the cell. Scale bars, 10 μm.

**Fig 5:**
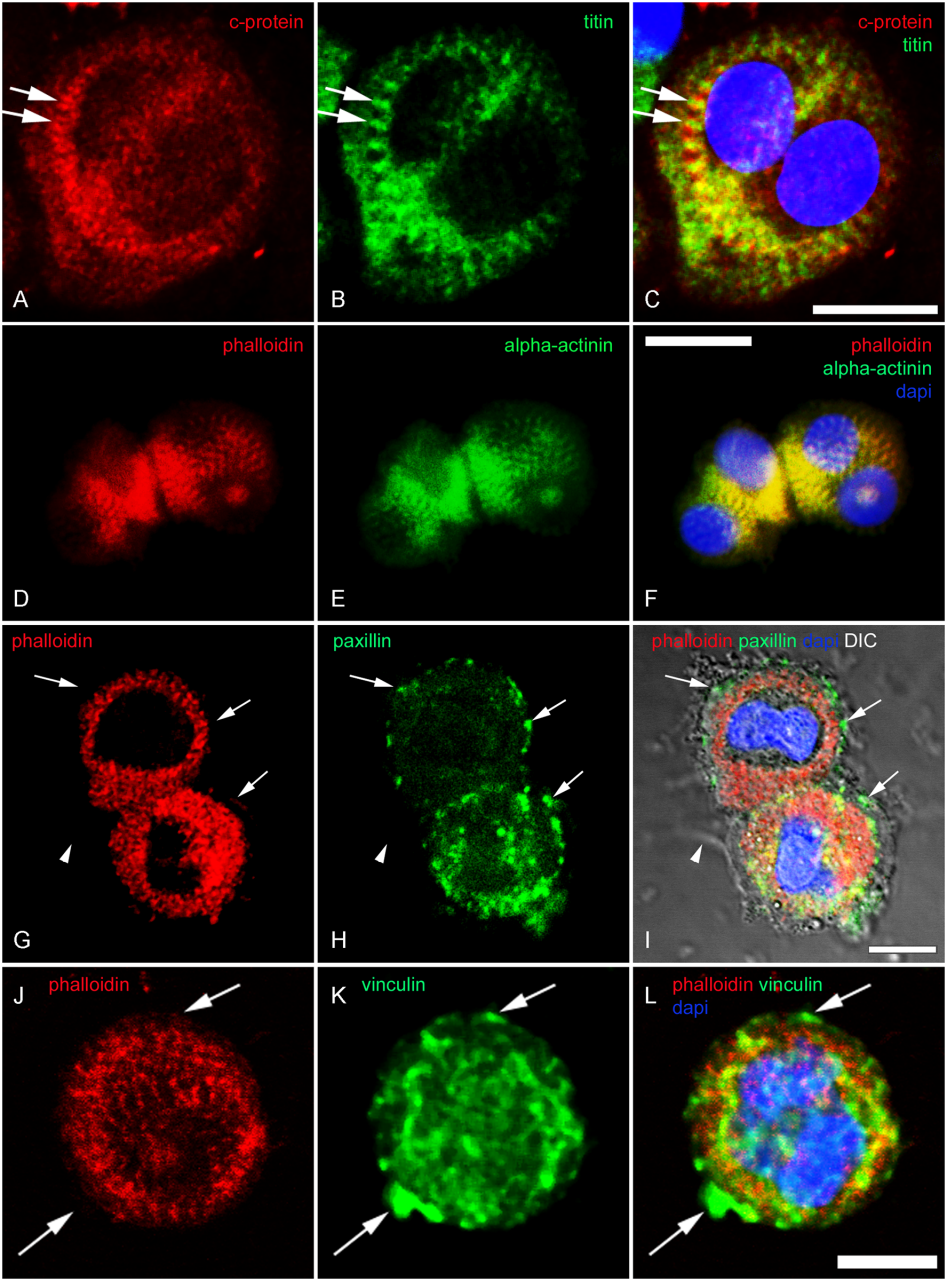
No breakdown of myofibril structure by long term CB treatment in myogenic cells. Confocal microscopy images of 72 hrs CB treated chick skeletal muscle cells are shown (**A**-**L**). Cells were stained with antibodies against C-protein (red in **A** and **C**), M-line titin (green in **B** and **C**), Rho-phalloidin (red in **D**, **F**, **G**, **I**, **J** and **L**), s-α-actinin (green in **E** and **F**), anti-paxillin (green in **H** and **I**), and anti-vinculin (green in **K** and **L**). Figures **C**, **F**, and **L** are superimposed images of **A**-**B**, **D**-**E** and **J**-**K**, respectively. In the case of **I**, DIC image was also merged with **G** and **H**. DAPI is shown in blue (**C**, **F**, **I** and **L**). Double arrows in **A**, **B** and **C** point to the complementary striations of C-protein and titin. Arrows in **G**, **H** and **I** show that myofibrils are restricted to the core of the cell whereas adhesion plaque protein clusters localized to the periphery of the cell. Arrowheads in **G**, **H** and **I** indicate a cytoplasmic extension that can be seen using DIC. Note the clusters of vinculin located outside the myofibrils (arrows in **J**, **K** and **L**). Scale bars, 10 μm.

**Fig 6:**
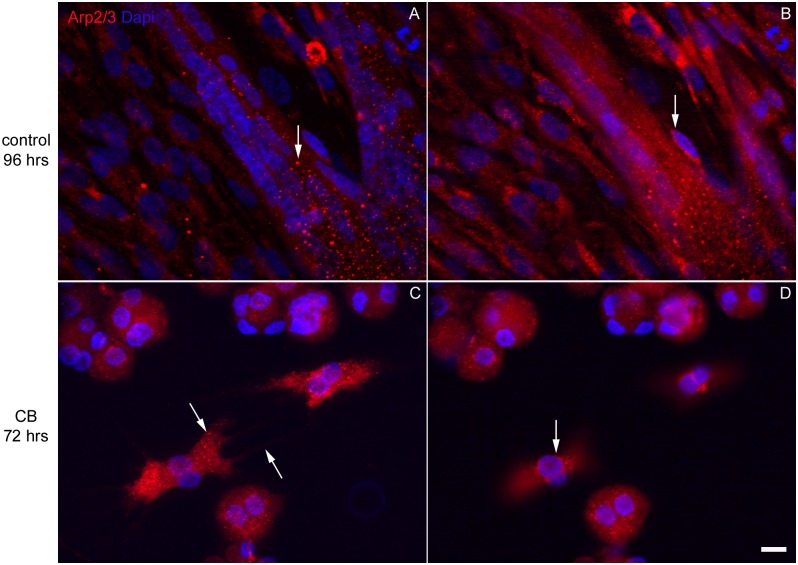
Changes in the distribution of the Arp2/3 complex in chick myogenic cells after long term CB treatment. Confocal microscopy images of chick myogenic cells treated on day 2 with CB for 3 days are shown (**C-D**). Some cultures were not treated (**A-B**). Cells were probed with antibodies to Arp2/3 (red in **A-D**) and DAPI (blue in **A-D**). Images **A** and **C** are Z-projections and images **B** and **D** are selected single focal plane slices. Figures **A-D** are merged images of the antibody staining combined with DAPI. Arp2/3 was detected in many cytoplasmic puncta of control-untreated myoblasts, fibroblasts and myotubes (arrow in **A**), as well as in the perinuclear region of cells (arrow in **B**). In CB-treated fibroblasts and myoblasts Arp2/3 was found in puncta in the cytoplasm (arrow in **C**) and at the perinuclear region (arrow in **D**). No Arp2/3 was found in the membrane extensions of the finger-shaped fibroblasts (right arrow in **C**). Scale bar, 10 μm.

Given the multiple functions attributed to the actin cytoskeleton, it was important to determine how cells lacking such a system for long time, due to the disruptive effects of CB, would function. Seventy-eighty percent of the cells in day 4 cultures grown in the presence of CB for the last 72 hrs (n = 100 cells from 3 different experiments) were multinucleated (Fig 3). They were differentiated into two distinct phenotypes in approximately even numbers. They were the giant spread finger-shaped fibroblasts and giant globular myogenic cells. The first class of giant multinucleated cells was the finger-shaped fibroblasts (Figs 3 and 4, S2–S5 Figs). The characteristics of these bizarre cells was summarized as follows: (1) over 90% harbor 2 to 5 nuclei at their cell center, which under the confocal microscope measured > 15 μm in the Z-axis (S5 Fig); (2) they had several finger-like protrusions, which varied from 3 to 10 (Fig 4 and S2 Fig). The individual fingers varied from 2 to 7 μm in the x-y axis and from 2–5 μm in the z-axis (S5 Fig); (3) recognizable subplasma F-actin cortices, stress fibers, adhesion plaques or ruffled membranes were absent; (4) the scattered, F-actin patches vary in size and often were > 100 in number (S5 Fig). Interestingly, they were not crystalloid (*e*.*g*. Fig 3); (5) as the antibodies to the 4 adhesion plaque proteins (paxillin, β1-integrin, vinculin and talin) diffusely stained in the entire cell, many actin patches were obscured. But from those that were visualized ~90% double stain with paxillin, whereas we estimated ~60% double stained with β1-integrin, vinculin and talin. Furthermore, the four adhesion plaque proteins assembled a thin, widely distributed, filigree network not observed in control-untreated fibroblasts (Fig 4B and 4C); (6) over 70% of the fingers terminated in lamellipodia of varying sizes. Many of the larger ones exceeded 20 μm in the x-y axis (Figs 3 and 4). Visually we estimated that ~90% of the total F-actin per cell was sequestered in their giant lamellipodia (Fig 4D–4H). Intriguingly, (a) unlike lamellipodia in diploid control fibroblasts, these lamellipodia were positive for all 5 of the tested proteins (α-actinin, paxillin, talin, β1-integrin and vinculin), and (b) these lamellipodia did actively translocate; (7) filopodia and ruffled membranes were conspicuously absent; (8) every finger-fibroblast was vimentin positive, but negative for all myofibrillar proteins including desmin (S4 Fig).

**Fig 7:**
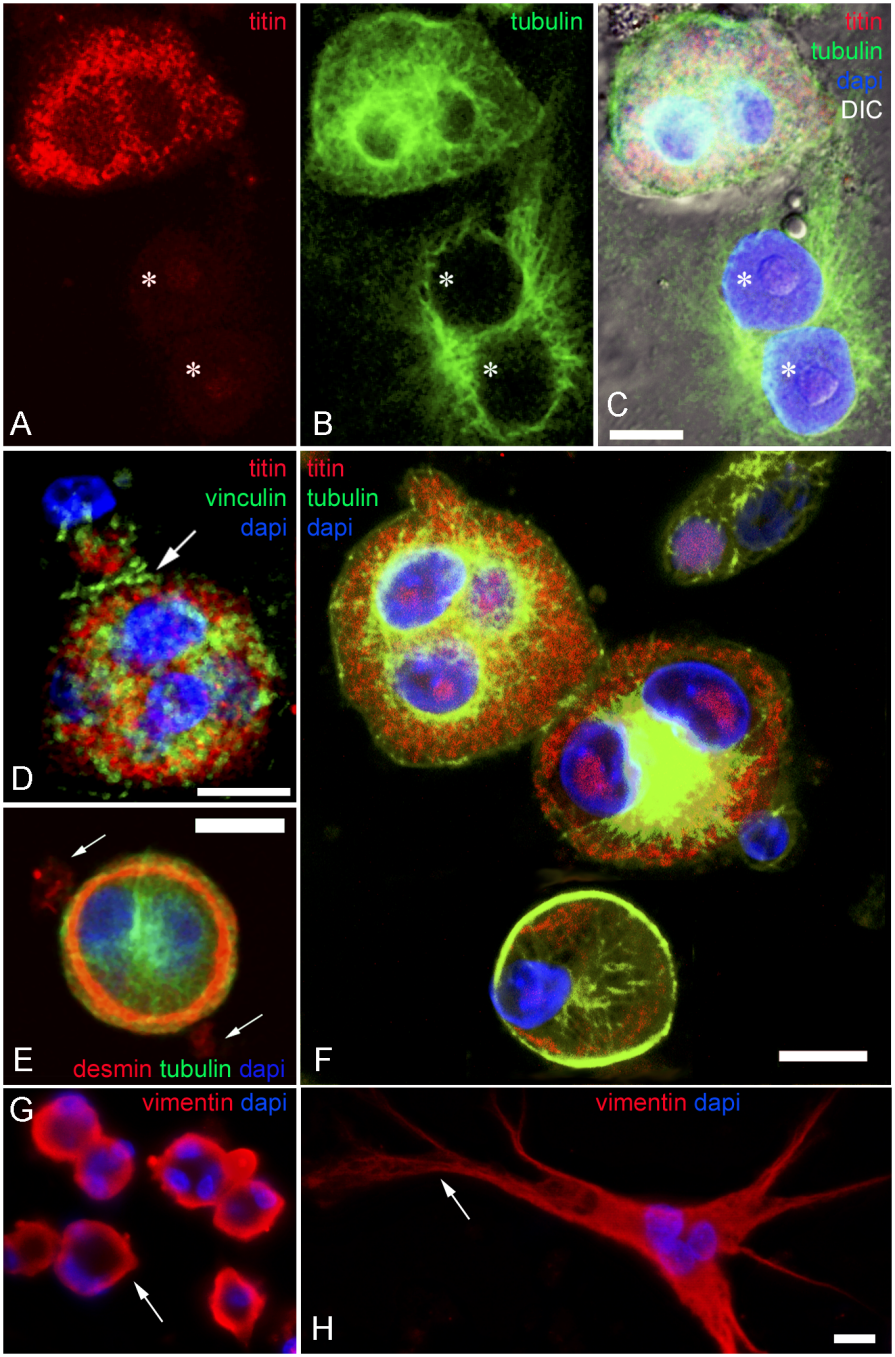
Abnormal localizations of desmin, vimentin and microtubules in long term CB treated myogenic cells. Confocal microscopy images of chick myogenic cells treated on day 2 with CB for 3 days are shown (**A**-**F**). Cells were stained with antibodies against Z-band titin (red in **A**, **C**, **D** and **F**), tyrosylated-α-tubulin (green in **B**, **C**, **E** and **F**), vinculin (green in **D**), desmin (red in **E**) and vimentin (red in **G** and **H**). Figure **C** is a superimposed image of **A** and **B** combined with DIC. Figures **D**, **E** and **F** are merged images of the double antibody staining. Nuclei are stained with DAPI (blue in **C**, **D**, **E**, **F, G** and **H**). Asterisks in **A**-**C** point to the flat fibroblast nuclei. Arrow in **D** shows the accumulation of vinculin in the region where two cells touch each other without fusing. Note the desmin localization in the cortex of a round cell and in cell extensions barely visible in this focal plane (indicated by arrows in **E)**. Condensed microtubule bundles were occasionally observed as a ring along round cell cortex (green in **F**). Vimentin was detected in the cortex of round myoblasts (arrow in **G**) and spreading in the whole cytoplasm of finger-shaped fibroblasts (**H**). Arrow in **H** point to vimentin filaments at cytoplasmic extension. Scale bars, 10 μm.

In contrast to finger-shaped fibroblasts, mushroom-shaped globular myogenic cells (Figs 5–7, S4 and S6 Figs) which contained mono-, bi-, or 4^+^-nuclei were myofibrillar protein positive (Fig 5). 70% of these globular-shaped myoblasts contained 2 or more nuclei. These myogenic cells also occasionally contracted spontaneously (S7 Fig). Long term CB treatment induced the loss of stress fiber like structures in these cells. However, polarized 1.0 μm α-actin containing thin filaments, which were assembled into normal I-Z-I-bands interdigitating with normal A-bands, were observed (Fig 5 and S6 Fig), indicating that CB did not interfere with the assembly of myofibrils but did interfere with their elongation mechanisms. Furthermore, CB treatment blocked cell fusion since we observed ~65% of the round-shaped muscle cells closely contacted with each other but unfused (Figs 5D–5I and 7D). Most of the cell surface of the rounded myogenic cells did not connect to the substrate (S3, S4 and S6 Figs), and there were aggregates of adhesion proteins around the surface of the cell and in the cytoplasm. We found both α-actin and s-α-actinin positive patches at the surface of peripheral sarcolemma of the rounded myogenic cells. These actin-containing patches were positive for focal adhesion proteins (Fig 4B and 4C), suggesting that focal adhesion proteins were closely associated with α-actin.

Since we detected actin structures (F-actin patches) with phalloidin in both fibroblasts and muscle cells after long term CB treatment (72 hrs), we decided to analyze the distribution of Arp2/3 proteins, which are known to be involved in the nucleation of actin monomers from existing filaments [8–11]. Arp2/3 was detected in many cytoplasmic puncta and in the perinuclear region of both myoblasts and fibroblasts that were not treated with CB (arrow in Fig 6B). In multinucleated myotubes from control-untreated cultures (72 hrs) Arp2/3 was found in many bright cytoplasmic puncta (arrow in Fig 6A). After 72 hrs of CB treatment Arp2/3 was found in puncta in the cytoplasm and at the perinuclear region of round-shaped myoblasts and finger-shaped fibroblasts (Fig 6C and 6D). Interestingly, almost no Arp2/3 was found in the membrane extensions of CB-treated finger-shaped fibroblasts (arrow in Fig 6C).

Finally, to investigate microtubules and intermediate filaments after long-term CB treatment, finger-shaped fibroblasts and in round myogenic cells were visualized with anti-tubulin and anti-desmin antibodies (S4 Fig). Both cell types were positive for tubulin, but in different distributions (S3 and S4 Figs). In fibroblasts, numerous microtubules were found and they were long, softly bended, spreading within the whole cytoplasm and radiating from the cell center at the nuclear vicinity towards the distal tip of each finger-shaped cytoplasm projection (Fig 4E and 4G, S3 and S5 Figs). MTOCs were clearly seen in these fibroblastic cells (Fig 4G and 4H, and S3 Fig). In round-shaped myoblasts, microtubules were shorter, spreading the whole cytoplasm and concentrated around the nuclear membrane and forming a ring line along the border of the rounded cells (Fig 7E and 7F, and S3 Fig). No visible MTOC or microtubules radiating centers were seen. Condensed microtubules accumulated at the peripheral region of the cells were observed in addition to the normal network of microtubules (Fig 7A–7C and 7F). Quantification of the number of myoblasts that present these structures showed that 15% of long-term CB treated myoblasts displayed condensed microtubules in their cytoplasm (n = 100 cells from 3 different experiments, significantly different from untreated cultures, p<0.05). Furthermore, intermediate filament desmin was detected spreading in the whole cytoplasm of round-shaped myoblasts and concentrated in ring structures which located between the myofibrils and condensed microtubule bundles (Fig 7E, and S4 Fig). Quantification of the number of round muscle cells displaying desmin rings showed that 7% of all desmin positive cells from 72 hrs CB-treated cultures present ring structures located at the periphery of the cells (n = 100 cells from 3 different experiments, significantly different from untreated cultures, p<0.05). Finger-shaped fibroblasts were negative for desmin (S4 Fig). Vimentin intermediate filaments were detected in 72 hrs CB-treated cultures in the cortex of round-shaped myoblasts (arrow in Fig 7G) and spreading in the whole cytoplasm of finger-shaped fibroblasts (Fig 7H). Vimentin filaments were clearly observed at the cytoplasmic extensions of finger-shaped fibroblast cells (arrow in Fig 7H). Vimentin labeling in CB-treated myoblasts resembles the desmin positive-ring structures found at the periphery of round shaped myoblasts (compare Fig 7E and 7G), suggesting a possible colocalization of these two intermediate filament proteins. Changes in the distribution of microtubules and intermediate filament structures after long-term CB treatment may be caused by the mechanical modifications induced by the disassembly of actin networks. It is important to point out that we found an extensive network of microtubules and intermediate filaments in these chick myogenic cells after long-term treatment with CB, i.e., without a detectable network of actin filaments.

Since CB is dissolved in DMSO, we decided to investigate the possible effects of DMSO alone after long-term treatments in these chick myogenic cell cultures. Phase contrast microscopy shows that cultures treated with DMSO for 72 hrs were indistinguishable from control-untreated cultures grown for the same period of time (S8 Fig). In both conditions (untreated-control and DMSO-treated cultures) it is possible to see the formation of long multinucleated myotubes (S8 Fig). In contrast, cultures treated with CB for 72 hrs show no myotube formation, and instead show many finger-shaped fibroblastic cells and globular-shaped myoblasts (S8 Fig).

### Recovery of actin cytoskeleton in 72 hrs-CB treated fibroblasts

**Fig 8:**
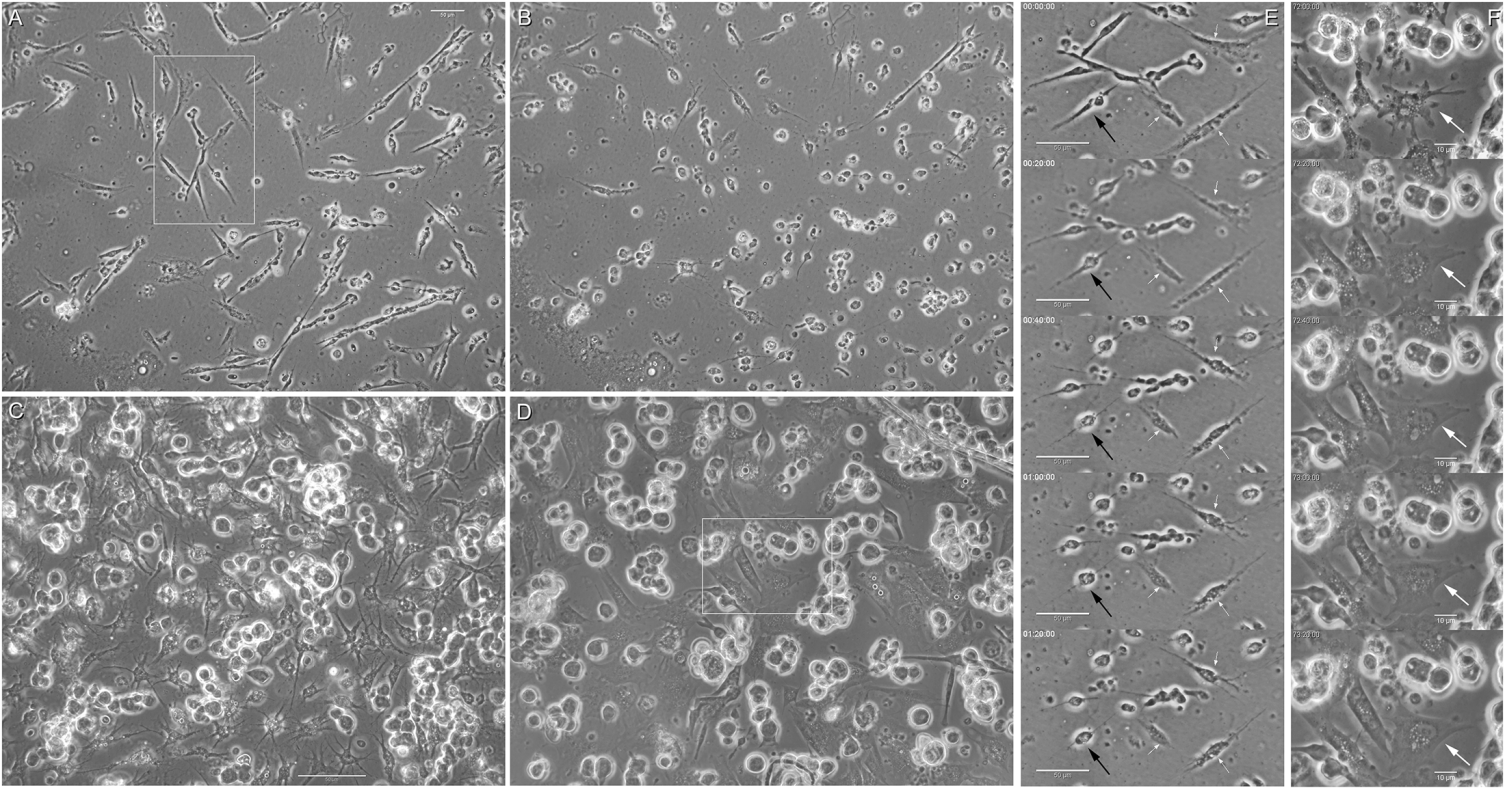
Cell shape changes during CB treatment and recovery of muscle cells and fibroblasts. Phase contrast microscopy images of chick myogenic cultures 24 hrs after cell plating shows thin and elongated myoblasts and flatten fibroblasts (**A**). After 30 min of CB treatment, both myoblasts and fibroblasts became rounded (**B**). After 72 hrs of CB treatment, giant finger-shaped fibroblasts and mushroom-shaped myoblasts can be seen (**C**). During the initial 20 min of recovery after 72 hrs of CB treatment (**D**), myogenic cells remain globular shaped whereas finger-shaped fibroblasts loses their finger protrusions and changes into flatten multinucleated spread cells (white arrows in **F**). These changes can be better appreciated in the time lapse sequences shown in **E** (30 min CB treatment) and **F** (recovery from 72 hrs of CB treatment). Each frame from top to bottom corresponds to a 20 min interval (time interval is shown in the top left of each image). Images shown in **E** and **F** are enlargement from the insets shown in images **A** and **D**, respectively. Note in **E** that both muscle cells (black arrows) and fibroblasts (small white arrows) become round-shaped after 30 min of CB treatment. Scale bars, 50 μm in A-E and 10 μm in F.

**Fig 9:**
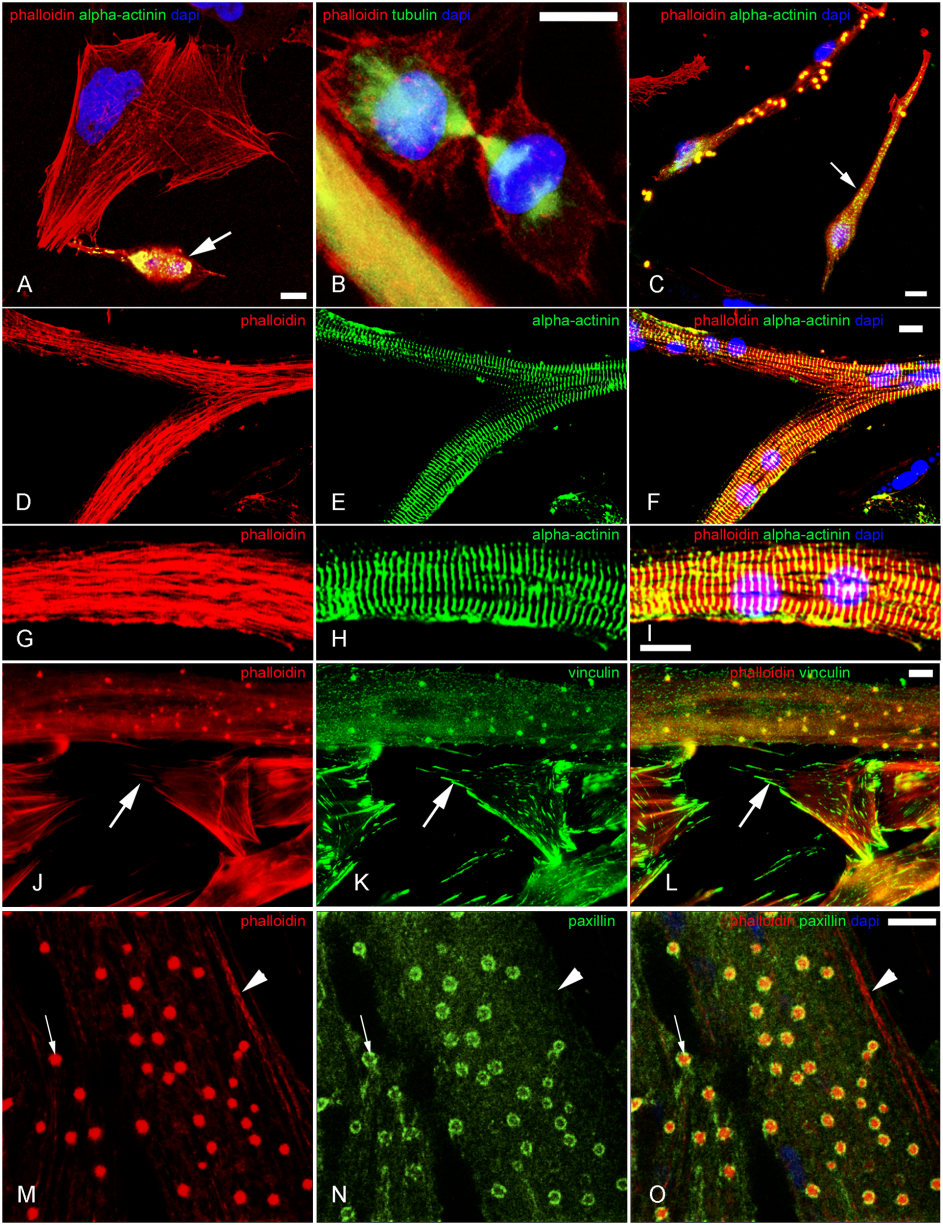
Recovery of fibroblasts and muscle cells from long term CB treatment. Confocal microscopy images of chick myogenic cells treated on day 2 with CB for 3 days and recovered for 1 day in normal media (**B**, **D-O**) or in the presence of cycloheximide (**A** and **C**) are shown. Cells were stained with Rho-phalloidin (red in **A**, **B**, **C**, **D**, **F**, **G**, **I**, **J**, **L**, **M** and **O**), antibodies against s-α-actinin (green in **A**, **C**, **E**, **F**, **H** and **I**), tyrosylated-α-tubulin (green in **B**), vinculin (green in **K** and **L**) and paxillin (green in **N** and **O**). Images **A**, **B** and **C** are merged images of the phalloidin and antibody staining. Images **F**, **I**, **L** and **O** are superimposed images of **D**-**E**, **G**-**H**, **J**-**K** and **M**-**N**. Nuclei were stained with DAPI (blue in **A**, **B**, **C**, **F**, **I** and **O**). Arrows in **A** and **C** point to myoblasts. Image **C** shows a minimal number of sarcomeres in the recovery of CB in the presence of cycloheximide (arrow). Focal planes are different between images **J-L** and images **M-O** were taken at a focal plane close to the substrate. Adhesion plaque structures are positive for vinculin but negative for Rho-phalloidin (arrows in **J**, **K** and **L**). Arrows and arrowheads in **M**-**O** indicate CAB and cortical actin in myotubes, respectively. Scale bars, 10 μm.

As soon as washed off CB and shifted to normal medium for 20 min, finger-shaped fibroblasts gradually extended their membrane tension, resulting in eliminating finger protrusions, *i*.*e*. the morphology of fibroblasts changed from finger-shaped fibroblasts to flatten multinucleated spread cells with immense cytoplasm (Fig 8F and S7 Fig). During the initial 20 min of recovery after 72 hrs of CB treatment, it is possible to observe under phase-contrast microscopy the disappearance of membrane protusions at the periphery of the fibroblastic cells (arrows in Fig 8F and S7 Fig). Vinculin emerged as dots at the edge of membrane and then gradually formed adhesion plaques where stress fibers start/end. During recovery, not only the spatiotemporal sequence of actin containing structures in the finger-shaped fibroblasts was different from recovery in the 30 min CB-treated diploid cells, but also the transitory structures displayed in the recovery of 30 min CB were not observed in the recovery of 72 hrs CB cultures. The actin cortex did not reconstitute precociously. Rather, even after a 60 min CB-washout, most cells tended to be circular, circumscribed by a ~2.0 μm fringe (S7 Fig). Structurally the fringe displays features more reminiscent of lamellipodia than ruffled membranes. During this period virtually all actin patches disappeared. Modest numbers of incipient adhesion plaques and poorly defined stress fibers appeared to be in the process of being nucleated and assembled at the periphery of the cell. Reconstitution and integration of all the subunits of the former finger-fibroblasts required 4–6 hrs (Fig 9A, 9B and 9J–9L). To examine whether *de novo* synthesized proteins were required for fibroblast recovery from long-term CB treatment, cycloheximide (a protein synthesis inhibitor) was added to recovery medium (Fig 9A). Fibroblasts treated on day 2 with CB for 3 days and recovered in the presence of cycloheximide showed sub-plasma membrane actin cortices and multiple bundles of actin containing stress fibers that were undistinguished from control-untreated cultures and from long-term CB treated cultures allowed to grown in normal media without cycloheximide (Fig 9B). These results suggest that newly synthesized proteins are not necessary for the formation of stress fibers during the recovery of fibroblasts from CB treatment.

### Slow recovery of muscle cells from 72 hrs CB exposure

In contrast to relatively quick response of fibroblasts to the removal of CB, myogenic cells recovered very slowly. During the initial 20 min of recovery after 72 hrs of CB treatment, myogenic cells remain globular shaped (Fig 8F and S7 Fig). It took about 24 hrs to form elongated myotubes from globular myogenic cells. We observed that the α-actin patches disappeared and then were replaced with a veil-like sarcolemma surrounding the shaft of myotubes, which was also observed with phalloidin labeling (Fig 9J). Myofibril structures were still intact during recovery process (Fig 9D–9I). In time-lapse videomicroscopy it was possible to observe that the mushroom-shaped myogenic cells contract spontaneously during treatment and recovery from CB treatment (S7 Fig), which is in accordance with our results showing that these globular and multinucleated cells contain intact and well organized myofibrils (Fig 5 and S6 Fig).

**Fig 10:**
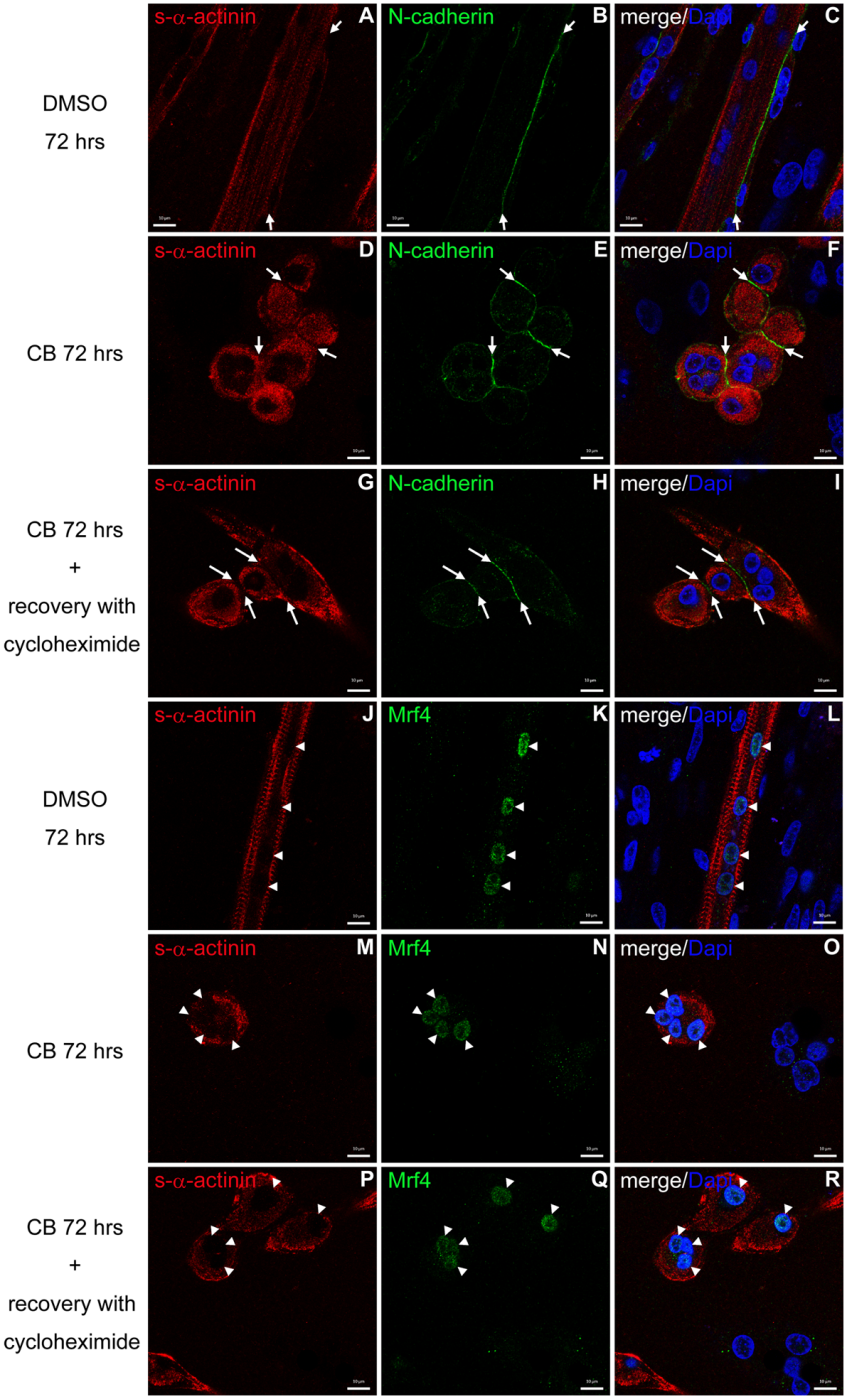
Distribution of N-cadherin and Mrf4 in myogenic cells that were treated with long-term CB and subsequent recovery in the presence of cycloheximide. Confocal microscopy images of myogenic cells which were 72 hrs DMSO- (A-C, J-L), 72 hrs CB-treated (D-F, M-O), or recovered from 72 hrs CB treatment in the presence of cycloheximide (G-I, P-R). Cells were stained with antibodies against s-α-actinin (red in A, C, D, F, G, I, J, L, M, O, P and R), N-cadherin (green in B, C, E, F, H and I) and Mrf4 (green in K, L, N, O, Q and R). Nuclei were also stained with DAPI (blue in C, F, I, L, O and R). Figures of C, F, I, L, O and R, are merged images of the double antibody staining and the DAPI. Arrows in A-I point to N-cadherin positive structures which are located at cell-cell contact regions. Mrf4 are specifically located to myonuclei in s-α-actinin positive muscle cells (arrowheads in J-R). Scale bars, 10 μm.

To examine whether *de novo* synthesized proteins were required for myotube recovery from long-term CB treatment, cycloheximide was added to recovery medium (Fig 9A and 9C). Muscle cells grown in the presence of cycloheximide elongate but fail to fuse, and show a minimal number of sarcomeres (Fig 9C). As cell adhesion molecules mediate the recognition and adhesion to promote cell fusion [22], we examined whether or not N-cadherin was localized correctly during recovery from CB in the presence of cycloheximide. Anti-N-cadherin antibody specifically captured their signals at cell-cell contact regions of myogenic cells which were recovered from 72 hrs CB treatment in the presence of cycloheximide (Fig 10A–10I). These results confirm that CB treated muscle cells grown in the presence of cycloheximide elongate but fail to fuse. To further investigate whether the myogenic program proceeds or not during the recovery of CB in the presence of cycloheximide, cells were probed with an antibody against Mrf4, one of the myogenic regulatory factors [23]. The Mrf4 signal was detected in myonuclei during recovery from CB in the presence of cycloheximide (Fig 10J–10Q). These results suggest that newly synthesized proteins are necessary for the formation of a mature multinucleated myofiber during the recovery from CB treatment.

One of the most characteristic features during recovery was the appearance of cortical actin bodies (CABs) in the shaft of some myotubes at 1–6 hrs after washing out CB. We found that these CAB structures consisted of a core that was positive for both α-actin/s-α-actinin and a rim containing focal adhesion plaque proteins (Fig 9J–9O and S9 Fig).

## Discussion

### Distinct phenotypical impact of long-term CB treatment on fibroblasts versus muscle cells

**Fig 11:**
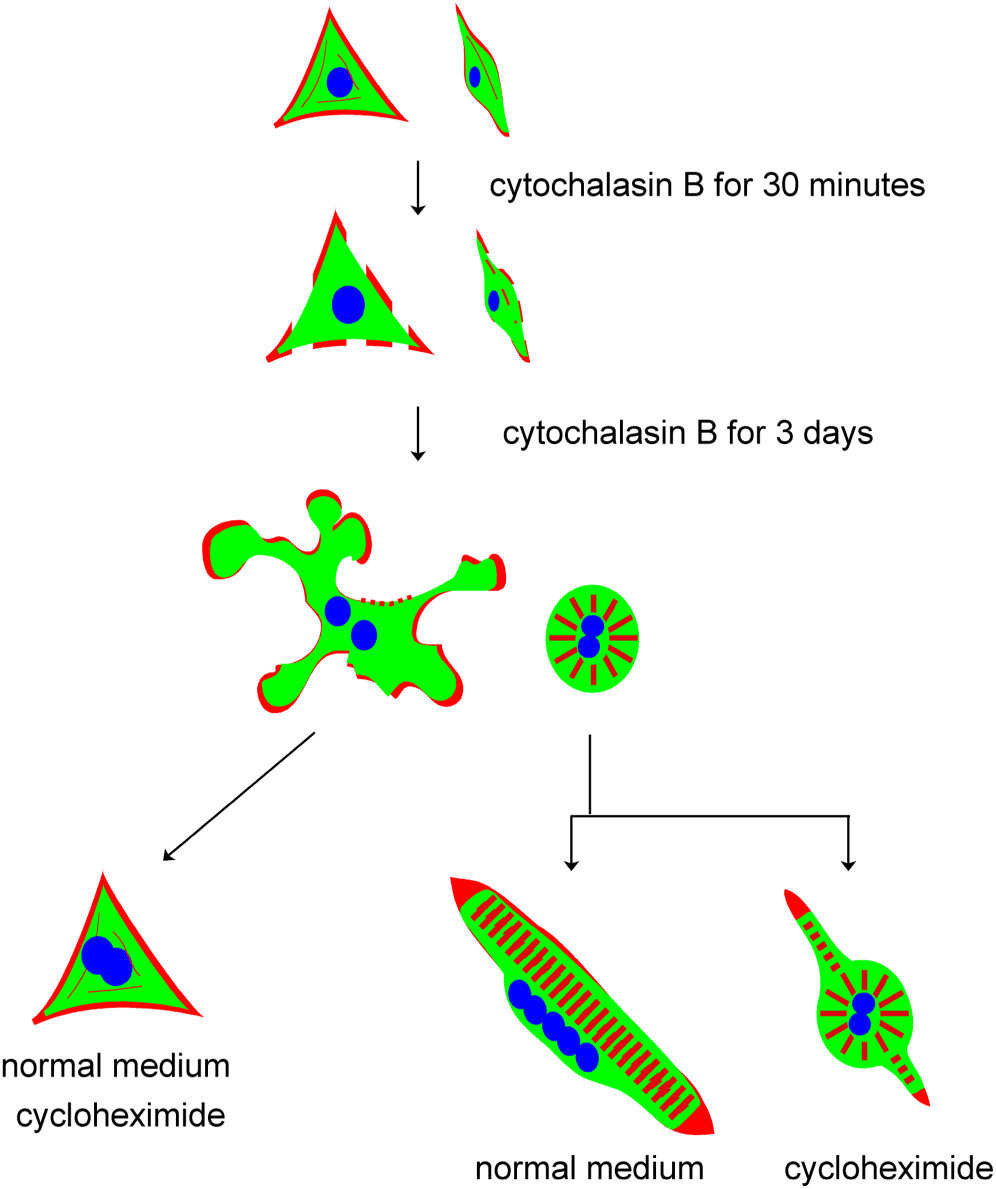
Scheme of distinct CB effects on different cell types. Fibroblasts and myoblasts treated for 30 min with CB lose their cortical actin and stress fibers in fibroblasts and stress fiber-like structures in myogenic cells. If treated for 72 hrs with CB, fibroblasts became giant multinucleated finger-shaped fibroblasts which contain ruffled membranes with actin on the tips, while myoblasts became multinucleated and round but perfectly striated. When recovered in normal medium, fibroblasts reassume their normal shapes, and muscle elongates and fuses to form myotubes. If recovered in the presence of cycloheximide, muscle cells elongate but fail to fuse and show a minimal number of sarcomeres. Red indicates actin-containing structures, green indicates cytoplasm and blue indicates nuclei.

After short-term CB treatment, the cell shape of both fibroblasts and myogenic cells become globular with very thin cytoplasmic extensions, resulting in indistinguishable cell shape. By contrast, long-term CB treatment led to distinct morphological impact on fibroblasts and muscle cells, *i*.*e*. finger-shaped fibroblasts and mushroom-like myogenic cells (Fig 11). Interestingly, most myoblasts and fibroblasts became multinucleated after long-term exposure to CB, with an even number of nuclei. This observation leads to the conclusion that CB blocks cytokinesis and myoblast fusion.

The morphology of the CB-treated fibroblasts is very dramatic: they are immense cells, almost 100 μm in diameter, with long processes and lamellipodia. This finger-shaped fibroblastic cell size and shape has never been described, particularly as an effect of CB treatment. The long-term effects of CB on myoblasts are quite different from the effects on fibroblasts. In fibroblasts CB disassembles stress fibers and adhesion structures preserving lamellipodia, whereas in muscle cells the cortical actin cytoskeleton is affected and the myofibrillar cytoskeleton is resistant to CB. After 72 hr CB treatment, myoblasts displayed striated myofibrillar structures that were positive for all the components of the sarcomere: thin filaments and Z lines (actin, tropomyosin, troponin, α-actinin), thick filaments and M-lines (C-protein, myosin heavy chain), and elastic filaments (titin and nebulin). These results differ from previous experiments, which suggested that CB induces the appearance of round myoblast cells containing only a few intact sarcomeres in chick myogenic cell cultures [24]. Our data shows that, even after long term CB treatment, myoblasts contain normal I-Z-I-bands interdigitating with normal A-bands. These data suggest two possibilities: (i) that CB did not interfere with the assembly of myofibrils, and/or (ii) that the myofibrils were preexisting and stable throughout the time course of CB treatment.

Even though most of the cell surface in the rounded CB-myoblasts does not connect to the substrate, there are aggregates of adhesion proteins around the surface of the cell and in the cytoplasm. The morphological differences between muscle and fibroblasts after long-term CB treatment may be caused by the presence of active lamellipodia in finger-shaped fibroblasts but not in mushroom-like myogenic cells. In normal fibroblasts, lamellipodia is a mobile component that drives the cell body [25] in which actin filaments are closely associated with the final ends of microtubules. We observed that almost all cytoplasmic extensions of finger-shaped fibroblasts are terminated with lamellipodia, which are very locomotive even in the presence of CB. We also show that a bundle of microtubules run from the cell body to the final tip of lamellipodia in finger-shaped fibroblasts. These results suggest that mobile lamellipodia followed by microtubules drives the cytoplasm in different directions, resulting in finger-shaped fibroblasts. Since lamellipodia are absent in mushroom-like muscle cells, they maintain a round shape after long term CB treatment.

### Different effects of CB on actin-containing structures

Actin-containing filament structures, such as stress fibers, adhesion plaques and cortical actin networks, are disorganized after CB treatment in muscle cells and fibroblasts. CB binds to the barbed end of actin filaments and inhibits the polymerization of actin filaments, resulting in breakdown of actin filament structures [19]. Phalloidin still, however, detects actin structures in both CB treated fibroblasts and muscle cells, indicating that actin molecules are still polymerized into actin oligomers in lamellipodia in fibroblasts and in actin patches in both cells. It is possible that actin oligomers, which consist of less than 10 actin molecules [26], are left behind in the CB treatment and are still polymerized at lamellipodia. As CB primarily caps and fragments long actin filaments into short oligomers, both aggregates in myoblasts and giant lamellipodia in finger-shaped fibroblasts should consist of densely packed oligomers.

To test our hypothesis that actin oligomers are left behind after long-term CB treatment and are still polymerized at lamellipodia, we used latrunculin B in CB treated myogenic cultures to prevent the polymerization of actin oligomers. Latrunculin B binds to actin monomers and prevents their incorporation into actin polymer. Latrunculin B is preferred for short-term studies because it is gradually inactivated by serum [27]. It has been shown that calf serum substantially increases the stability of actin filaments [27]. Therefore, latrunculin B could not work during long-term CB treatment (72 hrs). To test the possible use of latrunculin B in long-term CB treatment, we treated chick myogenic cultures that were in the presence of CB for 72 hrs with 250 mM of latrunculin B using two different culture media composition: normal 8-1-0.5 medium (MEM containing horse serum and chick embryo extract) and MEM without horse serum and chick embryo extract. These experiments showed that myogenic cells grown with MEM without serum suffered and eventually die after 24 hrs, whereas cultures grown in the presence of 8-1-0.5 medium grown well and showed no effects of latrunculin B (CB treated cultures were identical to cultures treated with CB and latrunculin B). The problem here is that we cannot discard the possibility that latrunculin B had no effects in the inhibition of actin oligomers because of its inactivation by the presence of serum in the medium.

Arp2/3 complex was found in both untreated and CB-treated chick myogenic cultures. Changes in Arp2/3 distribution was found after long term CB treatment, i.e., the absence of Arp2/3 labeling at the membrane extensions in finger-shaped fibroblasts. Since Arp2/3 complex is a nucleator of actin polymerization [8–11], the presence of a high quantity of Arp2/3 puncta in the cytoplasm of both fibroblasts and muscle cells after long term-CB treatment could be caused by an increase in Arp2/3 synthesis as a response to the absence of F-actin structures in these cells. New experiments are needed to confirm this hypothesis. It is important to point out that, to our knowledge, our data represents the first description of the cellular distribution of Arp2/3 proteins in skeletal muscle cells and during skeletal muscle differentiation.

The failure of adhesion plaque proteins to assemble into discrete focal adhesions but instead assemble into long multimolecular filaments, which is termed filigree, is of interest. In normal fibroblasts, focal adhesion plaques are formed behind lamellipodia and capped with stress fibers to regulate cell motility [28]. Our results show that CB treatment to fibroblasts diminished focal adhesion plaques but their components were partially localized at their lamellipodia, indicating that proper focal adhesion plaque complex is not formed in finger-shaped fibroblasts. It is clear that the breakdown of F-actin cytoskeleton induced by CB does not block the continued synthesis of the individual adhesion plaque proteins and that they assemble into ectopic long filament filigree, which is not observed in control-untreated cells. This filigree structure which runs through cytoplasmic extensions in finger-shaped fibroblasts is probably synthesized as a result of the absence of stress fibers, since F-actin stress fibers are essential to initiate the organization of proper focal adhesion plaques in fibroblasts [28].

The distribution of phalloidin-positive structures in chick fibroblasts briefly exposed to CB was identical to those reported by Ballestrem and colleagues [29] on living mouse melanoma cells (B16F1), Chinese hamster ovary cells (CHO), thymic endothelial cells, and 3T3 fibroblasts expressing EGFP/β-actin cDNA and exposed to CB, with one intriguing exception. Chick fibroblasts in CB do not form the conspicuous perinuclear actin rings or “actin clouds” they observed in mouse melanoma cells. Finding that actin containing structures in different cell types or in different structures within the same cell, respond differently to CB is of considerable interest.

Interestingly, we observed changes in microtubules and intermediate filament structures after CB treatment. The fact that changes in one cytoskeletal component can affect, more or less directly, all the cytoskeletal components suggest that there is a much deeper and subtle interaction between each part of the cytoskeleton than just direct molecular interaction as it has been repeatedly shown by sequencing of genes and identification of putative binding sites for several molecules. The changes observed in microtubules and intermediate filaments after CB could be caused directly by the modifications induced in the actin network or indirectly by other molecules. Further studies are necessary to understand the molecular machinery involved in the alterations of microtubules and intermediate filaments in myogenic cells in response to CB.

Given the evidence that the actin cytoskeleton serves as substrates for myosin motors, as well as in the distribution of mRNAs and relaying signals from the extracellular matrix to the nucleus, the finding that cells lacking this system nevertheless increase in mass, duplicate their chromosomes, and replicate their nuclei was unexpected. Even more challenging is how such cells when removed from CB retained mechanisms that permitted the re-expression of a relatively normal cell phenotype.

### Recovery from CB treatment

Long-term treatment with CB does not kill the myogenic cells, as demonstrated by their recovery capability. Instead, the cells respond to the stress by creating new morphologies using their cytoskeletons, depending on their genetic and differentiation state, *e*.*g*. CB-washout permits fusion into typical elongated myotubes with normal longitudinal striated myofibrils despite blocking fusion into myotubes in the presence of CB.

Our data showing the CB-induced actin disorganization and the CB recovery suggest that this system could be used to understand the roles of actin compartments in different cell functions, such as cell movement, cell fusion and cell adhesion. Association of actin, α-actinin, and the adhesion proteins in actin patches was observed when myogenic cells were exposed to CB for 72 hrs. This close correlation implies that the complexes play as the kernel to initiate the assembly of stress fibers or myofibrils during the recovery process. We could speculate that after CB washout the rapid reassemble of stress fibers in fibroblasts works in pre-existing short actin filaments rather than inducing *de novo* assembly of filaments, and that this process might be dependent on Rho GTPases [30].

Drastic rearrangements and inter-convertibility of the different actin cytoskeletal subunits are induced by a variety of extra- and intracellular signals. Rous sarcoma virus-transformed fibroblasts lose their global structures, which are replaced by numerous 1–2 μm multi-protein dot-like complexes [31]. These protein complexes, termed podosomes, accumulate at the membrane/substrate interface [32]. These podosomes closely resemble cortical α-actin containing bodies (CABs), which are formed in 12-O-tetradecanoylphorbol-13-acetate (TPA) treated myotubes [33]. Both podosomes and CABs consist of a core of actin and α-actinin surrounded by integrin, vinculin, talin and paxillin positive rims. As formation of CABs is thought to be an indicator of enhancing myofibrillar turnover [34], appearance of CABs reflects that myotubes intensively remodel their myofibrillar proteins during the recovery from CB. Furthermore, our result shows that newly synthesized myofibrillar proteins, including actin, are dispensable for the recovery from CB treatment. These data indicate the presence of a sufficient pool of pre-existing myofibrillar proteins to assemble a minimal number of sarcomeres but further protein synthesis is necessary to produce a mature myofiber.

It is important to point out that although CB has been shown to bind to the glucose transporter and interferes with cellular glucose uptake [35], as pointed out by the authors [35], it seems unlikely that the various other biological effects of CB, such as the inhibition of microfilament polymerization, cytokinesis, cell movement, or phagocytosis, can be attributed to the observed interference with the glucose transport system.

## Materials and Methods

### Primary skeletal muscle cell culture

The work was performed in accordance with the requirements of the Ethics Committee for Animal Care and Use in Scientific Research of the University of Pennsylvania, which specifically approved this study. Primary myogenic cells were prepared as described previously [36, 37]. Briefly, pectoral muscle tissue was removed from 11-day old chicken embryos (CBT Farms, Chestertown, MD). After separation of the connective tissue under a dissecting microscope, muscle tissue was minced with surgery knives and digested with trypsin (0.025%) at 37°C in a 5% CO_2_ incubator for 25 min. To stop digestion, a small volume of growth medium (10% horse serum, 10% chick embryo extract, 2 mM L-Glutamine, 50 U/ml penicillin, 50 μg/ml streptomycin, Fungizone in Eagle’s Minimum Essential Medium [Gibco]) was added to the sample and then centrifuged for 5 min at 800 rpm. After discard the supernatant, the pellet was suspended in the growth medium and filtered through lens paper to produce mononucleated cell suspension. Isolated mononucleated cells were plated onto collagen-coated Aclar coverslips (Pro-Plastics, Linden, NJ) at initial densities of 4.5 x 10^5^ cells per 35 mm culture dish. They were grown continuously in 1.5 ml of the growth medium. Cells were grown under humidified 5% CO_2_ atmosphere at 37°C.

### Drug treatments

To determine the short-term effect of CB on the actin disorganization, day 2 cultured cells were incubated with 5 μM CB in DMSO (both from Sigma-Aldrich) for 30 min. Some cells were treated with DMSO (2 μL) alone as a control. For recovery experiments, cells were washed with fresh growth medium 3 times, fed with fresh growth medium, kept in CO_2_ incubator for 30 min, and fixed. To determine the long-term effect of CB on the actin disorganization, day 2 cultured cells were incubated with 5 μM CB for 72 hrs. Some CB treated cells were recovered in the presence of the protein synthesis inhibitor cycloheximide (Sigma, dissolved in ethanol, working concentration 0.1 mM). Long-term CB dishes were pretreated with 0.1 mM cycloheximide for 1h before washout and then continue to recover in cycloheximide containing medium for designed time points. Some CB treated cultures were treated also with 250 mM of latrunculin B (Sigma-Aldrich). To analyze cell viability, trypan blue exclusion assays were performed in CB treated and non-treated cells. Cells were stained with 0.4% trypan blue and then examined microscopically to determine the total number of cells and trypan blue positive cells. The ratio of trypan blue positive cells was calculated as following: [(the number of trypan blue positive cells) / (the number of total cells)] × 100.

### Antibodies and fluorescent probes

The findings in this report rely heavily on the double-staining of cultured cells with Rhodamine (Rho)- or fluorescein (FITC)-phalloidin (1:40 and 1:10, respectively; Molecular Probes) and one of the following antibodies: anti-sarcomeric-α-actinin (s-α-actinin) antibody (1:2500, clone EA-53, Sigma), anti-β-actin (1:800, clone AC-15, Sigma), anti-titin Z1Z2 antibody (1:30, [38]), anti-C-protein (1:50, clone MF-1) that stains the A-bands of striated myofibrils [39], anti-nebulin M177M181 antibody (1:50, [40]), anti-desmin antibody (1:200, Sigma), anti-tyrosylated-α-tubulin antibody (undiluted, clone YL1/2), anti-vinculin antibody (1:50, clone VIN-11-5, Sigma), anti-paxillin antibody (1:50, BD Transduction Labs.), anti-β1-integrin antibody (1:10, Sigma), anti-talin antibody (undiluted, clone 8e6, Iowa Hybridoma Bank), anti-cadherin 2 (N-cadherin) antibody (1:5, BioGenex), anti-Mrf4 (Myf6) antibody (1:100, Santa Cruz Biotechnology), anti-Arp2/3 antibody (1:100, Abcam), and anti-vimentin antibody (1:100, clone Vim 3B4, Boehringer Mannheim).

### Immunofluorescence microscopy, image acquisition and processing

Indirect immunofluorescence double staining has been described by Lin and collaborators [36]. In brief, cultured cells, with or without drug treatment, were fixed with 2% formaldehyde in PBS for 3 min after washing with PBS at 37°C. Cells were permeabilized with 0.5% Triton X-100 in PBS for 10 min three times. For anti-tyrosylated-α-tubulin antibody staining, cells were fixed with 2% formaldehyde and 0.1% glutaraldehyde in PHEM (60 mM Pipes, 25 mM Hepes, 1 mM EGTA, 2 mM MgCl_2_, pH 6.95) for 10 min after washing with PHEM at 37°C. Fixed cells were permeabilized with 0.5% Triton X-100 in PHEM for 2 min. Subsequently, specimens were rinsed with 1 mg/mL sodium borohydride in PBS three times for 5 min and rinsed with PBS again to quench auto-fluorescence of glutaraldehyde. Specimens were incubated with primary antibody for 1 hour at 37°C. After washing with 0.5% Triton X-100 in PBS, specimens were incubated with secondary antibody for 1 hour at 37°C. Affinity-purified secondary antibodies conjugated with Rho or FITC were utilized (Jackson Immuno Research Labs.). To localize F-actin, some specimens were stained with Rho-phalloidin (3.3 μM, Molecular Probes Inc.). Cells were exposed to phalloidin for 20 min at 37°C. As a control for bleed-through artifact, all double-stained preparations were run in duplicate. In one series, each of the two primary antibodies was stained with either a Rho- or a FITC-conjugated secondary antibody. In the second series, the same primary antibodies were stained in the opposite fashion, thus each antigen was localized by both FITC- or Rho-tagged secondary antibodies. Nuclei were detected with 1 μg/mL DAPI (4, 6-diamidino-2-phenylindole dihidrochloride; Polyscience). Specimens were mounted in 90% glycerol in PBS containing 2.5% DABCO (1, 4-diazabicyclo (2, 2, 2) octane; Sigma). Cells were observed using a laser confocal microscope (LSM 510; Carl Zeiss), which employed a Zeiss Axiovert 100 inverted microscope. Images were viewed with a Zeiss C-Apo 63 x water immersion objective lens (NA 1.2). Rho, FITC and UV signals were visualized using appropriate lasers and filters. Image processing and stack projections were performed using ImageJ (http://imagej.nih.gov/ij/). Figure plates were mounted with Adobe Photoshop software (Adobe Systems Inc.). Some images were taken with a Leica DM model IRB fluorescence inverted optical microscope using 63 x 1.6 (NA 1.4) oil immersion objective lens.

### Videomicroscopy

Chick myogenic cells were grown for 24 hrs, treated with CB for 30 min or for 72 hrs. Live cultures were imaged under phase contrast microscopy in an Axiovert 100 microscope (Carl Zeiss, Germany). Images were recorded with an Olympus DP72 digital camera (Olympus, Japan) and analyzed using Fiji software.

### Statistical analysis

All data are represented as mean ± standard error. Statistical analysis was performed with one-way ANOVA on Ranks with Newman-Keuls Post Hoc test and statistical significance was defined as p<0.05.

## Supporting Information

S1 Fig Timelapse movie of cell shape changes during short term-CB treatment of muscle cells and fibroblasts. After 24 hrs of cell plating, embryonic chick myogenic cultures display thin and elongated myoblasts and flatten fibroblasts. After 30 min of CB treatment, both myoblasts and fibroblasts became rounded. Time is shown in the top left of each image. The movie was taken from the region shown in Fig 8A inset. Both muscle cells and fibroblasts become round-shaped after 30 min of CB treatment. Scale bar, 50 μm.
(AVI)

S2 Fig 3D reconstruction of cultured chick embryonic myogenic cells showing the morphological differences between finger-shaped fibroblasts and mushroom-like muscle cells after 72 hrs of cytochalasin B treatment. Sequential projections in the X and Y-axis from a stack of 1-μm interval images, acquired using a laser scanning confocal microscope. Cells were labeled with an antibody against α-tubulin. Both fibroblasts and myoblasts are multinucleated. Scale bar, 5 μm.
(AVI)

S3 Fig Sequential images in the Z-axis of chick myogenic cells showing the differences in the distribution of microtubules, and intermediate filament desmin and nuclei between finger-shaped fibroblasts and mushroom-like muscle cells after 72 hrs of cytochalasin B treatment. The 3-μm apart images were taken from a stack of 1-μm interval slices, acquired using a laser scanning confocal microscope. Cells were labeled with antibodies against α-tubulin (green) and desmin (red), and with DAPI (blue). Compare the spread and flat fibroblasts with the round and thick desmin-positive myoblasts. Note that the fibroblast nuclei are located in the 6 and 3-μm slices whereas the myoblast nuclei are present in all the slices. Scale bar, 10 μm.
(TIF)

S4 Fig 3D reconstruction of chick myogenic cells showing distinct morphological differences between a finger-shaped fibroblast and a mushroom-like muscle cell after 72 hrs of cytochalasin B treatment. Sequential projections in the X and Y-axis from a stack of 1-μm interval images, acquired using a laser scanning confocal microscope. Cells were labeled with antibodies against α-tubulin (green) and desmin (red), and with DAPI (blue). Note the desmin signal was detected at the periphery of the myoblast but not observed in the fibroblast. Microtubules are well spread in the fibroblast and concentrated in the center of the round myoblast. Both cells are binucleated. Scale bar, 10 μm.
(AVI)

S5 Fig Selected X-Z and Y-Z sections and selected slice of a confocal stack showing a finger-shaped fibroblast after 72 hrs of cytochalasin B treatment. The sections were reconstructed from a stack of 22 images with 1-μm interval, acquired using a laser scanning confocal microscope. Cells were labeled with Rho-phalloidin (red), an antibody against α-tubulin (green), and DAPI (blue). Note that actin accumulates at the tip of the fibroblast fingers (close to the substrate), whereas microtubules (that label most of the cell cortex) do not localize to the very extremity of the cell. Scale bar, 5 μm.
(TIF)

S6 Fig 3D reconstruction of a muscle cell after 72 hrs of cytochalasin B treatment. Sequential projections in the X and Y-axis from a stack of 1.4-μm interval images, acquired using a laser scanning confocal microscope. Cells were labeled with an antibody against sarcomeric-α-actinin (red) and DAPI (blue). Note that the round myoblast contains perfectly striated myofibrils, as seen in the Z-line staining of sarcomeric-α-actinin. The round myoblast has 4 nuclei. Scale bar, 5 μm.
(AVI)

S7 Fig Timelapse movie of cell shape changes during recovery of muscle cells and fibroblasts from long term-CB treatment. During the initial 20 min of recovery after 72 hrs of CB treatment, myogenic cells remain globular shaped whereas finger-shaped fibroblasts loses their finger protrusions and changes into flatten multinucleated spread cells. Time is shown in the top left of each image. The movie was taken from the region shown in Fig 8D inset. Scale bar, 10 μm.
(AVI)

S8 Fig DMSO shows no effects on myogenesis after long-term treatment of chick myogenic cell cultures. Phase contrast microscopy images of chick myogenic cells treated on day 2 with shows DMSO for 3 days are shown (**A-C**). Note that cultures treated with DMSO are indistinguishable from control-untreated cultures (compare **A** and **B**). In both conditions (control-untreated and DMSO-treated cultures) it is possible to see the formation of long multinucleated myotubes (**A** and **B**). Cultures treated with CB for 3 days show finger-shaped fibroblastic cells (indicated by white arrow in **C**) and globular-shaped myoblasts (indicated by black arrow in **C**). Scale bar, 20 μm.
(TIF)

S9 Fig Sequential images in the Z-axis of a chick myotube showing the different actin structures that are present during the recovery of cells after 72 hrs of cytochalasin B treatment. The images were taken from a stack of 1-μm interval slices, acquired using a laser scanning confocal microscope. Cells were labeled with Rho-phalloidin (red), an antibody against vinculin (green), and DAPI (blue). Actin is found in striated myofibrils at the dorsal part of the multinucleated myotube, whereas at the ventral surface of the cell actin (surrounded by vinculin aggregates) emerges as small and round cortical actin containing bodies (CABs). Scale bar, 5 μm.
(TIF)

## Abbreviations

CABs: Cortical α-actin containing bodies
CB: Cytochalasin B
DAPI: 4, 6-Diamidino-2-phenylindole dihidrochloride
DIC: Differential interference contrast
F-actin: Filamentous-actin
s-α-actinin: Sarcomeric-α-actinin
