# Supplementary figures and images for: Distinctive Effects of Cytochalasin B in Chick Primary Myoblasts and Fibroblasts
Source: PLoS One. 2016 Apr 27;11(4):e0154109. doi: 10.1371/journal.pone.0154109 (PMC4847871; doi:10.1371/journal.pone.0154109)

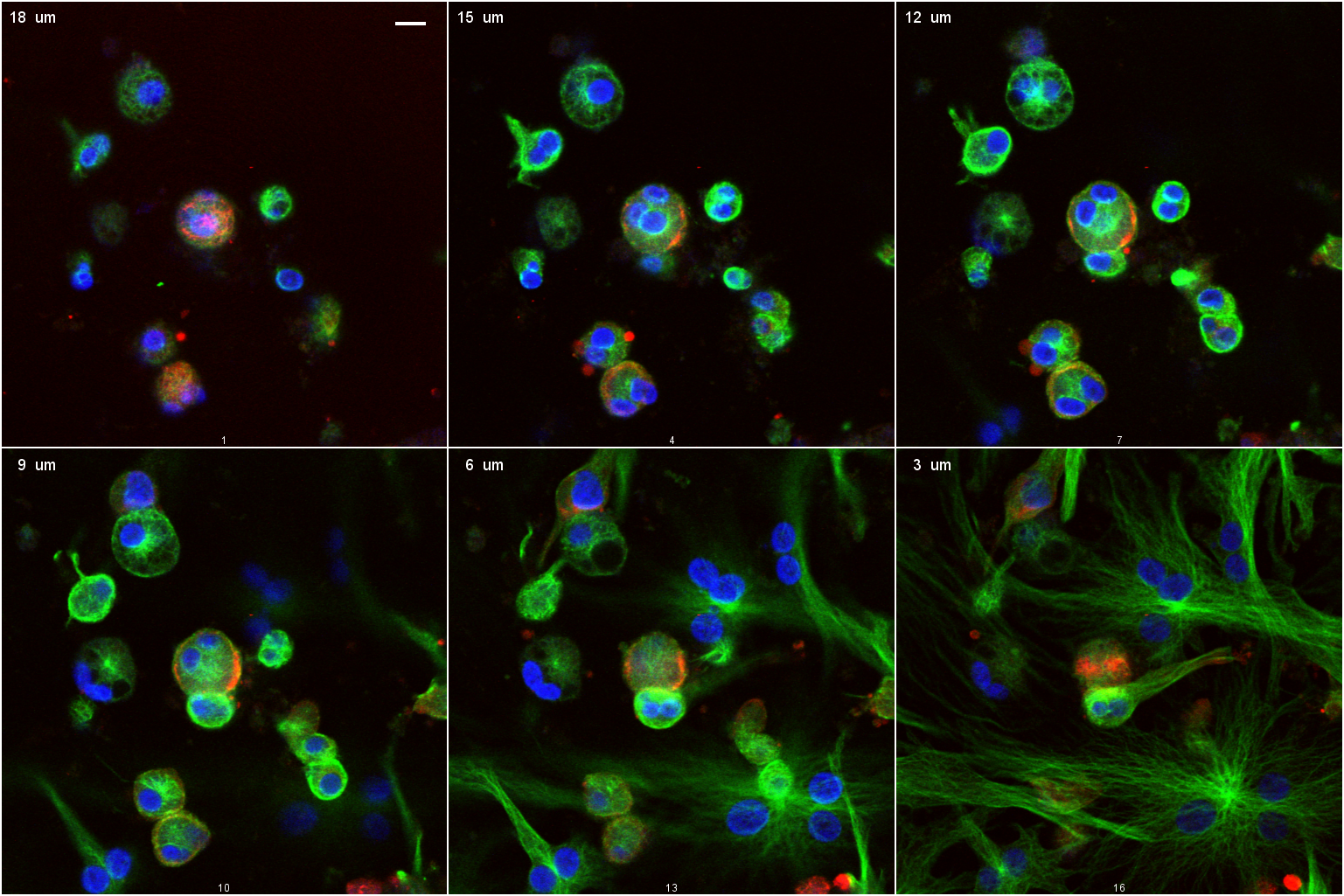

Supplement: S3 Fig — The 3-μm apart images were taken from a stack of 1-μm interval slices, acquired using a laser scanning confocal microscope. Cells were labeled with antibodies against α-tubulin (green) and desmin (red), and with DAPI (blue). Compare the spread and flat fibroblasts with the round and thick desmin-positive myoblasts. Note that the fibroblast nuclei are located in the 6 and 3-μm slices whereas the myoblast nuclei are present in all the slices. Scale bar, 10 μm. (TIF) [file pone.0154109.s003.tif]

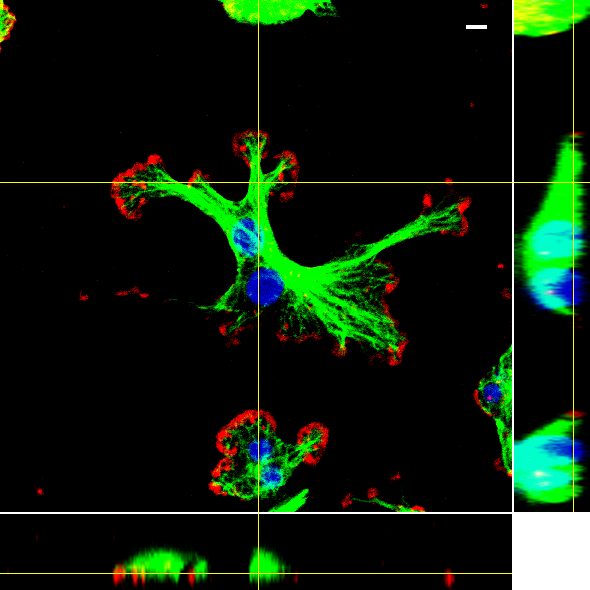

Supplement: S5 Fig — The sections were reconstructed from a stack of 22 images with 1-μm interval, acquired using a laser scanning confocal microscope. Cells were labeled with Rho-phalloidin (red), an antibody against α-tubulin (green), and DAPI (blue). Note that actin accumulates at the tip of the fibroblast fingers (close to the substrate), whereas microtubules (that label most of the cell cortex) do not localize to the very extremity of the cell. Scale bar, 5 μm. (TIF) [file pone.0154109.s005.tif]

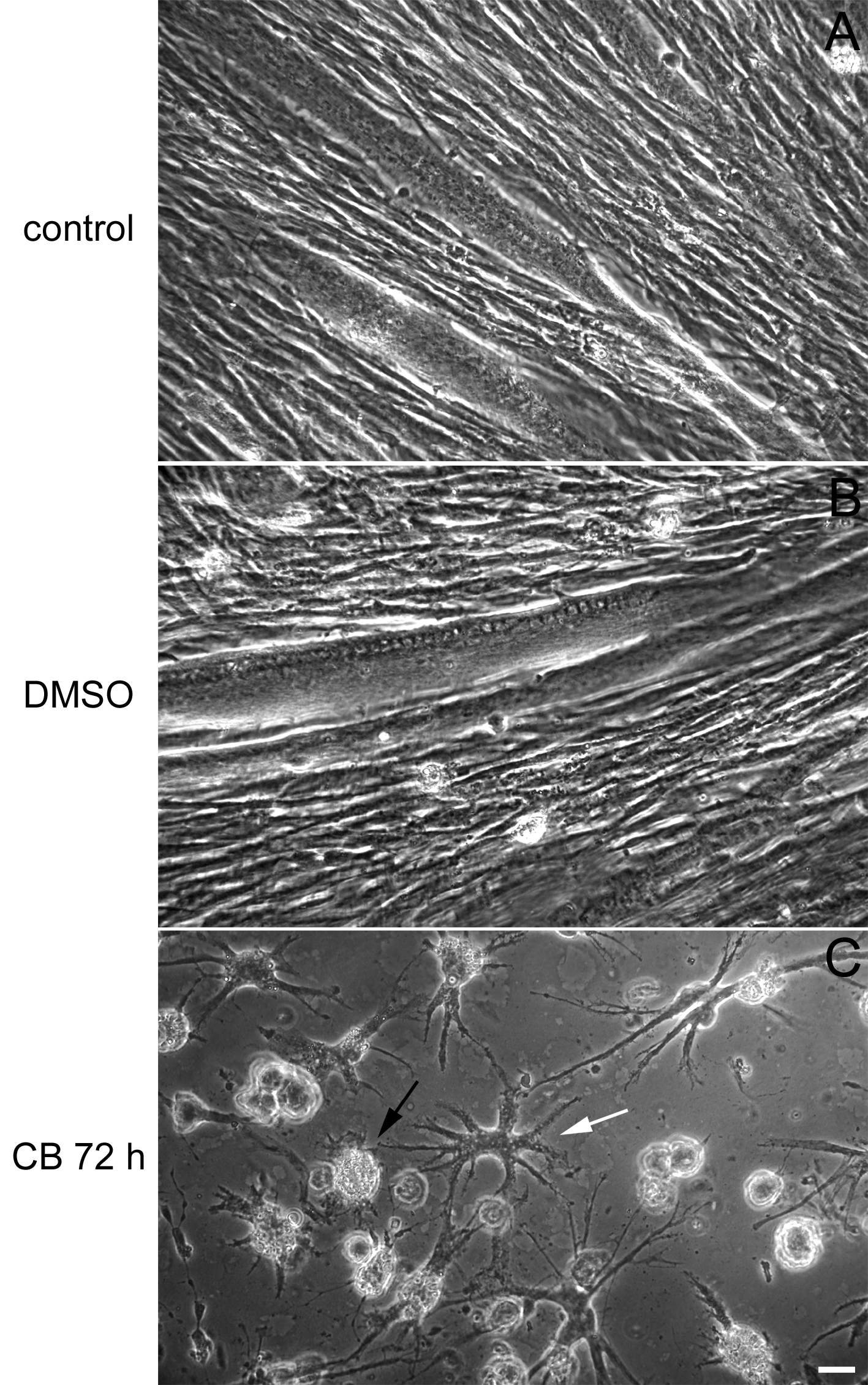

Supplement: S8 Fig — Phase contrast microscopy images of chick myogenic cells treated on day 2 with shows DMSO for 3 days are shown (A-C). Note that cultures treated with DMSO are indistinguishable from control-untreated cultures (compare A and B). In both conditions (control-untreated and DMSO-treated cultures) it is possible to see the formation of long multinucleated myotubes (A and B). Cultures treated with CB for 3 days show finger-shaped fibroblastic cells (indicated by white arrow in C) and globular-shaped myoblasts (indicated by black arrow in C). Scale bar, 20 μm. (TIF) [file pone.0154109.s008.tif]

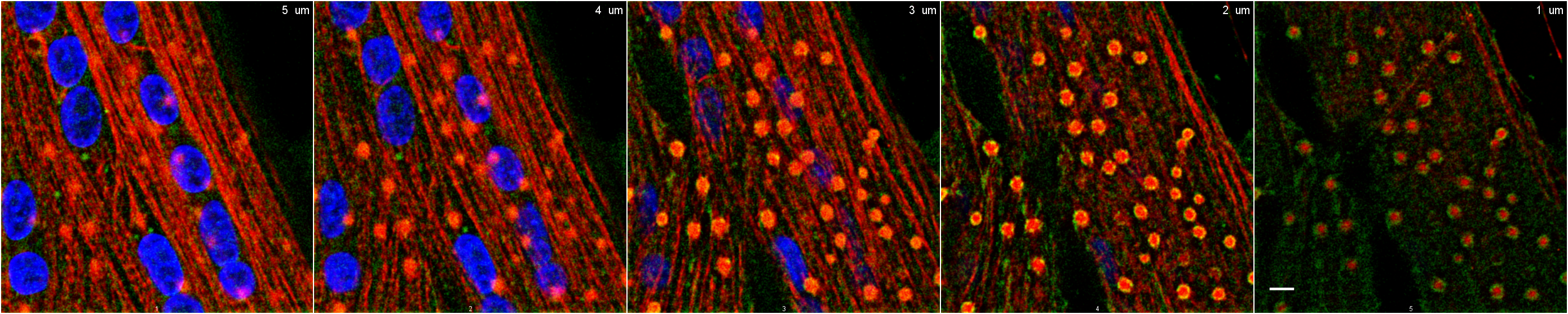

Supplement: S9 Fig — The images were taken from a stack of 1-μm interval slices, acquired using a laser scanning confocal microscope. Cells were labeled with Rho-phalloidin (red), an antibody against vinculin (green), and DAPI (blue). Actin is found in striated myofibrils at the dorsal part of the multinucleated myotube, whereas at the ventral surface of the cell actin (surrounded by vinculin aggregates) emerges as small and round cortical actin containing bodies (CABs). Scale bar, 5 μm. (TIF) [file pone.0154109.s009.tif]
